# The dynamic epigenetic atlas and its effects on instability of starch and seed storage protein quality traits in wheat (*Triticum aestivum* L.)

**DOI:** 10.3389/fpls.2025.1685120

**Published:** 2025-10-24

**Authors:** Yaping Wang, Lingfeng Miao, Haibin Wu, Xiaoliang Duan, Liu Chang, Yu Hong, Weilong Guo, Hui Sun

**Affiliations:** ^1^ Grain Quality and Nutrition Research Institute, Academy of National Food and Strategic Reserves Administration, Beijing, China; ^2^ Frontiers Science Center for Molecular Design Breeding, Key Laboratory of Crop Heterosis and Utilization, Beijing Key Laboratory of Crop Genetic Improvement, China Agricultural University, Beijing, China

**Keywords:** DNA methylation, histone modification, SSP, starch, wheat

## Abstract

Environmental factors significantly influence wheat grain quality by modulating starch biosynthesis and seed storage protein (SSP) composition, yet the underlying epigenetic mechanisms remain elusive. Here, we performed an integrated multi-omics analysis on the wheat cultivar ‘Fengdecunmai 5’ cultivated in two contrasting environments using WGBS, ChIP-seq (H3K27ac/H3K27me3), and RNA-seq. Epigenomic profiling revealed that environmental variation triggers extensive epigenetic reprogramming, with changes in H3K27ac enrichment being a primary epigenetic signature associated with the transcriptional divergence of starch and SSP biosynthesis genes during early development. Reconstruction of the transcriptional regulatory networks (TRNs) identified 86 essential transcription factors (TFs) that regulate 9,250 target genes in response to environmental impact. Our findings provide novel insights into the molecular basis of wheat quality instability across environments from an epigenetic perspective and highlight the critical role of histone modifications in regulating wheat seed quality.

## 1 Introduction

Wheat (*Triticum aestivum* L., AABBDD, 2n = 42) is cultivated at latitudes ranging from Scandinavia to Argentina. It is one of the most important food crops for human populations, contributing 20% of daily calorie intake and 15% of protein consumption. With its inherent quality attributes, wheat can be processed into a diverse array of products, such as bread, cakes, cookies, biscuits, pastries, and noodles. For breeders, the stability of quality characteristics is important as it impacts the selection efficiency. For the milling industries and bakers, the stability of raw material is crucial since it guarantees constant procedures and low product loss during processing (Grausgruber et al., 2000; Mut et al., 2010). However, the wheat grain processing traits vary with breeding cycles or local variations. To date, numerous studies have explored the impact of environmental factors on grain composition and quality characteristics, with their findings extensively documented in the literature (Awulachew, 2019; Bilgin et al., 2016; Dencic et al., 2011; Desheva, 2016; Eljak et al., 2018; Ghafoor et al., 2024; Hristov et al., 2010; Kaya and Akcura, 2014; Rozbicki et al., 2015; Thungo et al., 2020; Vázquez et al., 2012).

The end-use quality of wheat flour is predominantly determined by seed storage proteins (SSPs) and starch. SSP, primarily gluten protein, confers the unique viscoelastic properties of dough (Payne et al., 1987). The gluten proteins consist of monomeric gliadins and polymeric glutenins. Glutenins include high-molecular-weight glutenin subunits (HMW-GSs: 70–140 kDa) and low-molecular-weight glutenin subunits (LMW-GSs: 30–50 kDa) (Gao et al., 2010). It is generally agreed that both HMW-GS and LMW-GS are important in determining dough properties of bread wheat flours (He et al., 2005). According to the mobilities on electrophoresis at low pH, gliadins are traditionally divided into *α*-, *β*-, *γ*-, and *ω*-gliadins (Woychik et al., 1961). The globular gliadin plasticizes and acts as fillers in the glutenin polymeric network. Through their interactions with glutenins, gliadins contribute to dough viscosity (Uthayakumaran et al., 1999).

In addition to proteins, the physicochemical properties of starch also influence dough quality (Cao et al., 2019; Gao et al., 2020; Li et al., 2021, 2023; McCann et al., 2016; Roman et al., 2018; Zi et al., 2019). Starch usually consists of approximately 25% amylose and 75% amylopectin. The ratio between these two polymers affects starch properties such as gelatinization, pasting, and gelation (Sasaki et al., 2000; Zeng et al., 1997). Furthermore, the amylose/amylopectin ratio has been shown to influence dough and baking qualities (Hung et al., 2005; Lee et al., 2001; Morita et al., 2002).

To date, many transcription factors (TFs) involved in gluten gene regulation have been identified. For instance, storage protein activator (*SPA*) and *TaNAC019* activate the expression of glutenin genes (Albani et al., 1997; Conlan et al., 1999; Gao et al., 2021; Ravel et al., 2014). Additionally, *TaPBF-D*, *TaGAMyb*, and *TaFUSCA3* have been reported to enhance *HMW-GS* gene expression (Diaz et al., 2002; Guo et al., 2015; Sun et al., 2017; Zhu et al., 2018). Many starch biosynthesis-related genes have been identified, including *GBSS*, ADP-glucose pyrophosphorylase (*AGPase*), starch synthase (*SS*), branching enzyme (*BE*), debranching enzyme (*DBE*), and starch/*α*-glucan phosphorylases (*PHOs*) (Botticella et al., 2018). TFs are also involved in the regulation of starch synthesis by regulating starch biosynthesis-related genes, such as Rice Starch Regulator 1 (*RSR1*) (Liu et al., 2016), Basic leucine-zipper TF 28 (*TabZIP28*) (Song et al., 2020), and *TaSPA* (Guo et al., 2020), a TF that also regulates SSP synthesis and accumulation.

Recently, with the rapid decline in the cost of second-generation sequencing, several technologies have been developed for application to large-scale epigenetic mapping. For example, chromatin immunoprecipitation sequencing (ChIP-seq) (Johnson et al., 2007) enables simultaneous identification of key TFs, enhancers, and other regulatory elements; furthermore, other platforms that detect DNA-protein interactions, such as cleavage under targets and release using nuclease (CUT&RUN) (Skene et al., 2018) and cleavage under targets and tagmentation (CUT&Tag) (Kaya-Okur et al., 2019) have been subsequently developed. Meanwhile, various methods have been developed to study chromatin accessibility. For instance, Assay for Transposase Accessible Chromatin with high-throughput sequencing (ATAC-seq) has been developed to identify open chromatin regions, nucleosome positioning, and regulatory motifs (Buenrostro et al., 2013); another antibody-free method, chromatin sequencing (Chrom-seq), enables the identification of chromatin-associated RNAs that play regulatory roles in epigenetic events (Fan et al., 2024). Other available methods of DNA methylation analysis, such as Whole-genome Bisulfite Sequencing (WGBS) (Paun et al., 2019), Methylation-Sensitive Amplification Polymorphism Sequencing (MSAP-Seq) (Guarino et al., 2020), and MethylRAD (Wang et al., 2015) have been developed and applied to detect dynamic DNA methylomes. By combining emerging bioinformatics tools and specialized algorithms, the above-mentioned technologies are widely applied to study epigenetic mechanisms for crop improvement.

With the advancement of epigenetic sequencing technologies, comprehensive approaches integrating multi-layer data have been developed. These approaches aim to decipher the roles of histone modifications, DNA methylation, and chromatin accessibility, as well as to identify key regulators in wheat endosperm development (Chen et al., 2024; He et al., 2024; Pei et al., 2023; Zhang et al., 2024; Zhao et al., 2024, 2023; Zhou et al., 2022). However, very few studies have explored the epigenetic mechanisms underlying the instability of quality traits in the same wheat variety under different environmental conditions. Our previous experimental data revealed significant variations in the quality traits of the wheat cv. ‘Fengdecunmai 5’ (*Triticum aestivum* L.) across different planting environments. In this study, we used ‘Fengdecunmai 5’ wheat samples collected from Guoyang County, Anhui Province (designated as GY) and Baixiang County, Hebei Province (designated as BX) as experimental materials. By employing multi-omics technologies, we dynamically analyzed DNA methylation, histone modifications (H3K27ac and H3K27me3), and gene expression levels in grains at 8, 16, and 32 days post anthesis (DPA). Furthermore, these analyses were correlated with key grain traits, including storage protein synthesis and starch development. Our objective was to identify candidate epigenetic modification sites responsible for the instability of quality traits in ‘Fengdecunmai 5’ under varying environmental conditions. By focusing on starch- and storage protein-related epigenetic markers, we aimed to provide insights into mitigating wheat quality instability across diverse environments.

## 2 Materials and methods

### 2.1 Plant materials and growth conditions

Wheat (*Triticum aestivum* L., AABBDD, 2n = 42) cv. ‘Fengdecunmai 5’ was grown in the experimental fields at two locations: Guoyang (GY) County (33°29′42″ N, 116°12′38″ E), Bozhou City, Anhui Province, and Baixiang (BX) County (37°29′13″ N, 114°39′51″ E), Xingtai City, Hebei Province during the 2022 and 2023 growing seasons. The meteorological elements (precipitation, sunshine duration, and temperature) from ERA5, which cover the entire growth period in the two locations, were downloaded (https://www.mirror-earth.com/) and presented in **Supplementary Figure S1**. The flowering time (anthesis) of each individual floret was marked, and only grains from the middle spikelet were harvested to ensure that the seeds were collected at the same developmental stage. Seeds were sampled at 8, 16, 24, and 32 DPA and stored at –80°C for subsequent RNA-seq, WGBS, and ChIP-seq. It should be noted that due to the high impact of a “wheat-soaked persistent rainfall” event in the Huang-Huai-Hai Plain (Gao and Gao, 2023), the heavy precipitation in late May in the two fields of Anhui and Hebei Provinces resulted in pre-harvest sprouting. Consequently, the mature seeds were discarded in the present study.

### 2.2 Extraction of gliadins and glutenins

Gliadins and glutenins were extracted and separated from seeds at three different grain-filling stages (16, 24, and 32 DPA) as previously described (Zhang et al., 2014). For each stage, approximately 200 mg of seeds were ground in 500 μl of 50% (v/v) 1-propanol using a frozen grinder (JXFSTPRP-CLN, Jingxin, Shanghai, China). An additional 500 μl of 50% (v/v) 1-propanol was then added to the ground seeds for the extraction of the gliadin fraction. The sample was vortexed for 2 minutes and incubated at 65°C for 30 minutes, with shaking every 10 minutes. After centrifugation at 13,000 rpm for 10 minutes, 900 μl of the supernatant was collected.

The glutenin fraction was extracted from the insoluble material obtained in the previous step. The sample was washed twice with 1 ml of 50% (v/v) 1-propanol, the mixture was incubated at 65°C for 30 minutes and centrifuged at 13,000 rpm for 10 minutes, and the supernatant was discarded. After washing, the pellet was air-dried in a clean bench for 30 minutes. Subsequently, 250 μl of a solution containing 50% (v/v) isopropanol, 20% (v/v) 1 M Tris-HCl (pH 6.8), 30% (v/v) deionized water, and 1% (w/v) DTT was added to the dried samples. The mixture was vortexed for 2 minutes at RT and incubated at 65°C for 30 minutes, with shaking every 10 minutes. Next, 250 μl of a solution containing 50% (v/v) isopropanol, 20% (v/v) 1 M Tris-HCl (pH 6.8), 30% (v/v) deionized water, and 1.4% (v/v) 4-vinylpyridine was added. The samples were vortexed for 2 minutes at RT and incubated at 65°C for 30 min, with shaking every 10 minutes. After centrifugation at 13,000 rpm for 10 minutes, 400 μl of the supernatants were collected and mixed with 600 μl of pre-cooled acetone. The mixture was stored at –20°C overnight. The next day, the samples were centrifuged at 13,000 rpm for 10 minutes, and the supernatant was discarded. The gluten precipitate was washed twice with 1 ml of absolute ethanol, followed by centrifugation at 4°C at a speed of 13,000 rpm for 10 minutes. The extracted glutenins were dried at RT and dissolved in 350 μl of a solution containing 50% (v/v) acetonitrile, 50% (v/v) deionized water, and 0.5% (v/v) trifluoroacetic acid (TFA). Finally, the dissolved gliadins and glutenins were filtered through a 0.45 μm nylon filter (Teknokroma, Barcelona, Spain) for further chromatography analysis.

### 2.3 Reversed-phase high-performance liquid chromatography

Reversed-phase high-performance liquid chromatography (RP-HPLC) analysis was conducted as previously described (Chen et al., 2019) with some modifications. A Waters Alliance e2695 system equipped with an Agilent ZORBAX 300SB-C18 column (5 µm, 4.6 × 150 mm i.d., Agilent Technologies, Santa Clara, CA, USA) was used. The mobile phase consisted of two solvents: (A) water and (B) acetonitrile (ACN), both containing 0.06% (v/v) TFA. The elution gradient for glutenins was as follows: 0−2 min, 21% B; 2−52 min, from 21% to 53.5% B; 52−54 min, from 53.5% to 90% B; 54−57 min, from 90% to 21% B; 57−64 min, 21% B. The elution gradient for gliadins was as follows: 0−2 min, 25% B; 2−27 min, 25% to 50% B; 27−29 min, 50% to 25% B; 29−35 min, 25% B. The column temperature was maintained at 60°C, and the flow rate was set to 1 ml/min. The injection volume was 8 μl, and the effluent was monitored at 210 nm. The total amounts of HMW-GSs, LMW-GSs, and gliadins were estimated by integrating the corresponding RP-HPLC peaks in the chromatograms. Three biological replications were performed, and each sample was analyzed in duplicate.

### 2.4 Total starch and amylose starch content determination

For each stage (16, 24, and 32 DPA), approximately 200 mg of dried seeds were ground for starch separation and purification according to the method previously described (Chen et al., 2014). Starch content was determined using a Megazyme Total Starch (AA/AMG) Assay Kit (Megazyme, Bray, Wicklow, Ireland). Three biological replicates were performed for all stages. For amylose content, a Megazyme Amylose/Amylopectin Assay Kit (Megazyme, Bray, Wicklow, Ireland) was used, with three biological replicates performed for each test.

### 2.5 DNA extraction and sequencing

Genomic DNAs were isolated from the seeds of wheat ‘Fengdecunmai 5’. DNA library preparation was performed using the BGI Optimal DNA Library Prep Kit (BGI-Shenzhen, China). Subsequently, the final double-strand library products were denatured to generate single-strand DNA. The resulting library was sequenced on the Illumina Hiseq X-Ten PE150 platform with an insert size of approximately 300 bp.

To identify differentially expressed genes (DEGs) in ‘Fengdecunmai 5’ grown in the two experimental fields, seeds harvested at three grain-filling stages (8, 16, and 24 DPA) from both planting regions were sent to BGI-Shenzhen for RNA isolation, library preparation, and sequencing. Three biological replicates were conducted for each stage. All constructed paired-end RNA libraries (2 × 150 bp) were sequenced on the DNBSEQ-T7-PE150 platform at BGI-Shenzhen. Clean reads were obtained by filtering out low-quality reads using standard quality control with FastQC software.

To comprehensively elucidate the dynamic changes of DNA methylation during wheat grain development and their potential impacts on gene expression, starch synthesis, and storage protein accumulation in ‘Fengdecunmai 5’ at the two experimental fields, seeds harvested at 16 and 24 DPA from both fields were sent to Biozeron company (Shanghai, China) for WGBS, aiming to construct single-base-resolution DNA methylation profiles. Two independent biological replicates were conducted for each stage. Genomic DNA was isolated and sheared to 200–400 bp using a Covaris S220 Focused-ultrasonicator (Covaris, USA). After bisulfite conversion of the DNA fragments, methylated sequencing adapters were ligated, and the libraries were size-selected and PCR-amplified. The Accel-NGS Methyl-Seq DNA Library Kit (Swift, USA, Catalog #: 30096) was used to append sequencing adapter to the 3’ end of the fragments. Library quality was assessed on the Agilent 5400 system (Agilent, USA) and quantified via qPCR (1.5 nM). Finally, paired-end sequencing was performed on an Illumina NovaSeq™ X Plus system (Illumina, USA), yielding 200 Gb of data per biological replicate.

Furthermore, in order to examine potential regulatory roles of histone modifications in transcriptional activity, starch biosynthesis, and storage protein deposition in ‘Fengdecunmai 5’ at both experimental fields, we performed ChIP-seq on ‘Fengdecunmai 5’ seeds harvested at 16 and 24 DPA from the two experimental fields to profile the genome-wide dynamic distributions of H3K27me3 and H3K27ac, following the established protocol (Wang et al., 2016). Chromatin immunoprecipitation was performed using specific antibodies against H3K27me3 (Abcam, Cat. no. ab6002) and H3K27ac (Abclonal, Cat. no. A7253). Libraries were prepared with the KP201–02 Library Preparation Kit (TransGen Biotech) and sequenced on an Illumina NovaSeq 6000 platform (Annoroad Gene Technology, Beijing, China) in 150-bp paired-end mode.

### 2.6 Alignment and genomic variant calling

Raw reads were trimmed with Trimmomatic (Bolger et al., 2014) to remove adapters and low quality reads. The resulting high-quality clean reads were mapped to the Chinese Spring wheat reference genome (IWGSC RefSeq v1.0) (International Wheat Genome Sequencing Consortium (IWGSC), 2018) with BWA-MEM. Alignment files were then filtered using Bamtools (v2.4.1) (Barnett et al., 2011) to exclude reads with long insert sizes (>10,000 bp), negative insert sizes (<−10,000 bp), zero-length inserts (=0 bp), or low mapping qualities (<1). Potential PCR duplicates were removed using SAMtools (v1.3.1) (Li et al., 2009).

Variant calling was performed using the GATK (v3.8) (McKenna et al., 2010) HaplotypeCaller in GVCF mode. Preliminary filtering of single nucleotide polymorphisms (SNPs) was conducted using GATK VariantFiltration function with the following parameters ‘-filterExpression QD < 2.0 || FS > 60.0 || MQRankSum < −12.5 || ReadPosRankSum < −8.0 || SOR > 3.0 || MQ < 40.0 || DP > 30 || DP < 3’. For insertions and deletions (InDels), the filtering criteria were ‘QD < 2.0 || FS > 200.0 || ReadPosRankSum < −20.0 || DP > 30 || DP < 3’. SNPs and InDels that did not meet any of the additional following criteria were further excluded: (1) minor allele frequency ≥ 5%, (2) missing rate ≤ 40%, and (3) bi-allelic sites. Finally, the identified SNPs and InDels were annotated using SnpEff (v4.3) (Cingolani et al., 2012).

### 2.7 Processing of RNA-Seq data

Raw sequencing reads were quality-trimmed using Trimmomatic (Bolger et al., 2014) with the parameters ‘sliding-window: 4:20, minlen: 40’. The processed reads were then aligned to the Chinese Spring wheat reference genome (IWGSC RefSeq v1.0) (IWGSC, 2018) using STAR aligner (v2.7.8a) (Dobin et al., 2013) with default parameters. Uniquely mapped reads were selected using SAMtools (v1.11) (Li et al., 2009) with the parameter ‘−q 255’ for subsequent gene expression calculation. Uniquely mapped reads were quantified using featureCounts (v.1.5.2) (Liao et al., 2014) with default parameters, and expression levels were normalized to transcripts per million (TPM) values to account for transcript length and sequencing depth.

Given the dynamic and context-dependent nature of gene expression during wheat grain development, we employed a fold change (FC) threshold of 1.5 to identify differentially expressed genes (DEGs) using DESeq2 (v.1.16.1) (Love et al., 2014), as a slightly lower FC threshold helps avoid overlooking subtle but biologically relevant changes that may play roles in the regulatory networks under investigation. All DEGs were required to meet an adjusted *P*-value < 0.05. Furthermore, biological replicate correlations were calculated based on TPM values, and replicates with a minimum Pearson correlation coefficient (PCC) of 0.9 were retained. Genes with TPM ≥ 0.5 were defined as expressed genes.

### 2.8 Processing of WGBS data

Raw sequencing reads were processed using fastp (v.0.20.0) (Chen et al., 2018) with default parameters to remove low-quality reads. Clean reads were aligned to the reference genome using BS-Seeker2 (v.2.1.5) (Guo et al., 2013), with the following parameters ‘–aligner=bowtie2 –bt2–end to-end –bt2–very-sensitive –bt2–dovetail -m 1’. Methylation level quantification was subsequently performed using BS-Seeker2 with the parameters ‘–rm-overlap’.

To ensure accurate DNA methylation analysis, we excluded all cytosine-related SNPs and their flanking regions (± 3 bp). This is because mutations can disrupt methylation contexts, and heterozygous variants may obscure methylation state determination. Only sites with a read depth between 5 and 150 were retained for subsequent analyses.

Differentially methylated regions (DMRs) were identified using CGmapTools (v.0.1.2) (Guo et al., 2018) with the 200-bp sliding-window approach. The methylation level of each region was defined as the proportion of methylated cytosines (mC) relative to all observed cytosines (mC + unmethylated C). Mean methylation level for genomic windows was calculated and regions with minimum coverage thresholds (≥16 reads for CG/CHG, ≥64 reads for CHH) were retained in both samples. DMRs were defined as regions showing significant differential methylation (Fisher’s exact test, *P*_adj_ < 0.05) with absolute methylation differences exceeding 0.5 (CG), 0.3 (CHG), and 0.1 (CHH).

### 2.9 Processing of ChIP-seq data

Quality control was performed using fastp (v.0.20.0) (Chen et al., 2018) to filter out low-quality sequences. Cleaned reads were aligned to the wheat reference genome (IWGSC RefSeq v1.0) (IWGSC, 2018) using Bowtie2 (v2.3.5) (Langmead and Salzberg, 2012) with the parameters ‘–very-sensitive’ to accommodate variable fragment lengths and maximize sensitivity. Alignment files were subsequently processed using SAMtools (v1.11) (Li et al., 2009) for sorting and quality filtering (MAPQ ≥ 20), and Picard (v2.16.0) (http://broadinstitute.github.io/picard/) was used to remove clonal duplicates.

Peaks were called using MACS2 (2.1.4) (Zhang et al., 2008) with the following parameter ‘-f BAM, –keep-dup all, -g 1.7e10’. The R package ChIPseeker tool (v1.30.3) (Yu et al., 2015) was employed to annotate the peaks to the wheat genome. After merging peaks across all samples, the modification level of each peak in each sample was quantified by featureCount (v.1.5.2) (Liao et al., 2014). Differentially modified peaks (DMPs) between different samples were identified using DESeq2 (v.1.16.1) (Love et al., 2014), and peaks with adjusted q-value < 0.05 and log2 (fold-change)  ≥ 1 were classified as DMPs.

### 2.10 Real time quantitative polymerase chain reaction

For RT-qPCR, the reaction mixture was composed of 1 μL cDNA template, 0.6 μL each of forward and reverse primers (10 μM), 10 μL SYBR Green Mix (TIANGEN, FP217-02, Beijing, China), and the final volume was brought to 20 µL with RNase-Free ddH_2_O. Amplifications were carried out via nested PCR using a SensoQuest LabCycler (SensoQuest, Germany). Subsequently, quantitative PCR was performed on a CFX Connect™ Real-Time PCR Detection System (Bio-Rad, USA) using FastFire qPCR PreMix (TIANGEN, Beijing, China). The 2−ΔΔCt method was used to evaluate the expression level of the target gene, and the data were normalized to wheat *TaACTIN* (TraesCS5B02G124100). RT-qPCR was performed as technical triplicates per sample. Three biological replicates were performed. All primers are listed in **Supplementary Table S1** and were synthesized by Shanghai Sangon Biological Engineering Technology & Services Co., Ltd. (Shanghai, China).

## 3 Results

### 3.1 Dynamic changes in starch synthesis and protein accumulation in ‘Fengdecunmai 5’ across the two environments

**Figure 1:**
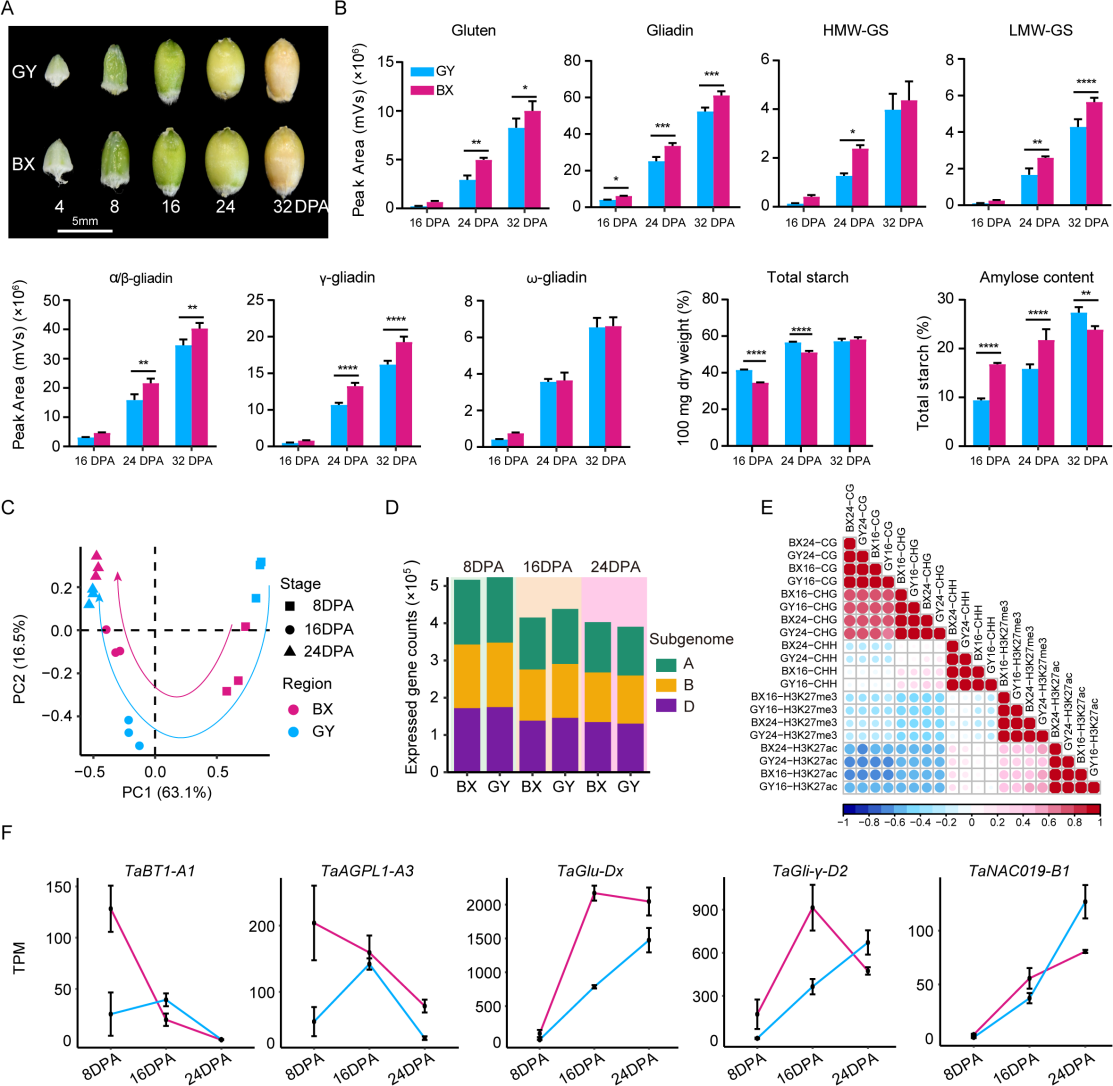
Field-dependent variations in starch, seed storage protein (SSP) content and ranscriptome and epigenome landscapes during ‘Fengdecunmai 5’ grain development. **(A)** Morphology of developing seeds at different stages. **(B)** Comparison of total starch content, amylose ratio, gliadin, glutenin, and their subunit contents in ‘Fengdecunmai 5’ grains at 16, 24, and 32 DPA between BX and GY. **(C)** PCA analysis of temporal RNA-seq data, with three biological replicates sequenced at each developmental time point. **(D)** Number of expressed genes (TPM > 0.5) in A, B and D subgenomes at 8, 16, and 24 DPA in BX and GY. **(E)** Scatter plots presenting the correlations among the densities of epigenetic marks across samples. **(F)** Comparison of gene expression changes of key SSP and starch biosynthesis genes at 8, 16, and 24 DPA of ‘Fengdecunmai 5’ grains between the two experimental fields.

To assess the environmental impact on wheat grain quality, we conducted a comparative analysis on the total starch content, amylose ratio, gliadin, glutenin, and their subunit contents in ‘Fengdecunmai 5’ grains at 16, 24, and 32 DPA from the two cultivation sites (**Figures 1A, B**). The results revealed that both starch and protein contents increased during grain development. The total starch content in ‘Fengdecunmai 5’ cultivated in GY was significantly higher than in BX at both 16 DPA and 24 DPA (*P* < 0.0001), though this difference became non-significant by 32 DPA. Conversely, the amylose ratio showed an opposite trend, with BX having significantly higher proportions at 16 DPA and 24 DPA (*P* < 0.0001) and GY exhibiting a higher ratio at 32 DPA (*P* < 0.01). Regarding storage protein accumulation, BX consistently demonstrated higher gliadin and glutenin contents across all developmental stages compared to GY, with significant differences (*P* < 0.01) observed in LMW-GS, α/β-gliadins, and γ-gliadins. These results indicate that ‘Fengdecunmai 5’ exhibited significant site-specific variations in starch and storage protein accumulation when cultivated at the two locations in 2022.

### 3.2 Global profiling of gene expression and epigenetic changes during grain development

To explore the epigenetic regulation in shaping wheat grain quality, we collected the grains from the middle spikelet of ‘Fengdecunmai 5’ at 8, 16, and 24 DPA, which were grown in BX and GY, and performed RNA sequencing, whole-genome bisulfite sequencing (WGBS), and chromatin immunoprecipitation coupled to parallel DNA sequencing (ChIP-seq) of two histone modifications, including acetylation of histone 3 at lysine 27 (H3K27ac) and tri-methylation of histone 3 at lysine 27 (H3K27me3). Overall, high-quality sequencing data were generated with an average of 15-fold coverage, with bisulfite conversion rates were > 99.5% for DNA methylomes, and mapping rates were > 93.2% for transcriptomes and > 89.89% for histone modification profiles (**Supplementary Table S2**). The biological replicates of the transcriptome (r > 0.9) showed high correlation for all stages (**Supplementary Figure S2**). The principal component analysis (PCA) revealed a similar developmental trajectory between GY and BX based on transcriptome data (**Figure 1C**). However, gene expression showed greater dynamics across 8, 16, and 24 DPA in GY compared to that in BX (**Figure 1C**), indicating a drastic effect of the environment on grain development. In addition, the number of expressed genes (TPM > 0.5) exhibited a decreasing trend during the seed developmental process in both experimental fields; compared to BX, GY showed a higher number of expressed genes at 8 and 16 DPA, while an opposite trend was observed at 24 DPA (**Figure 1D**).

Analysis of correlation coefficients revealed a strong positive association for the same type of epigenetic modification across different samples (r = 0.92–0.99) (**Figure 1E**). Additionally, CG methylation densities showed a high correlation with CHG methylation (r = 0.70–0.75). Interestingly, CHH methylation at 16 DPA exhibited a weak positive correlation with CHG methylation (r = 0.21–0.31) and was nearly uncorrelated with CG methylation (r = -0.08 to -0.04). In contrast, CHH methylation at 24 DPA was negatively correlated with CG methylation (r = -0.27 to -0.22) and showed no correlation with CHG methylation (r = 0.02–0.13) (**Figure 1E**), suggesting dynamic changes in DNA methylation during grain development. The density of the active mark H3K27ac was negatively correlated with both CG (r = -0.63 to -0.57) and CHG methylation (r= -0.53 to -0.51) but nearly uncorrelated with CHH methylation (r = -0.03 to 0.2). Unexpectedly, H3K27ac exhibited a moderately positive correlation with the repressive mark H3K27me3 (r = 0.36–0.59) (**Figure 1E**).

The expression changes of some key genes involved in SSP accumulation and/or starch biosynthesis during grain development were investigated (**Figure 1F**). For instance, the *TaBT1-A1* gene, which is positively correlated with starch synthesis, thousand-kernel weight, and grain width but negatively correlated with setback viscosity (Wang et al., 2019; Zhao et al., 2024), exhibited significantly higher expression levels in BX than in GY at 8 DPA. Similarly, the *TaAGPL1-A3* gene, which is positively related to AGPase activity and grain starch accumulation rate (Kang et al., 2013), also showed higher expression in BX at this stage. The expression levels of both genes increased at 16 DPA and then decreased sharply at 24 DPA in GY, and showed similar expression levels with that in BX. The expression of two other SSP synthesis genes, *TaGlu-Dx* and *TaGli-γ-D2*, which are positively related to HMW-glutenin and gliadin accumulation, respectively, increased progressively in GY during seed development (**Figure 1F**). In BX, their expression levels were comparable to GY at 8 DPA but increased to significantly higher levels than GY at 16 DPA. By 24 DPA, *TaGlu-Dx* expression showed a slight decrease though remaining elevated compared to GY, while *TaGli-γ-D2* expression declined sharply to levels below those in GY. The endosperm-specific TF *TaNAC019*, known to regulate glutenin and starch accumulation (Gao et al., 2021), demonstrated increasing expression in both fields, with GY showing higher levels than BX at 24 DPA (**Figure 1F**). These results suggested that differential expression of key genes involved in SSP accumulation and starch biosynthesis may contribute to the observed grain quality differences between BX and GY.

### 3.3 Reshaped transcriptome and epigenome during grain development

**Figure 2:**
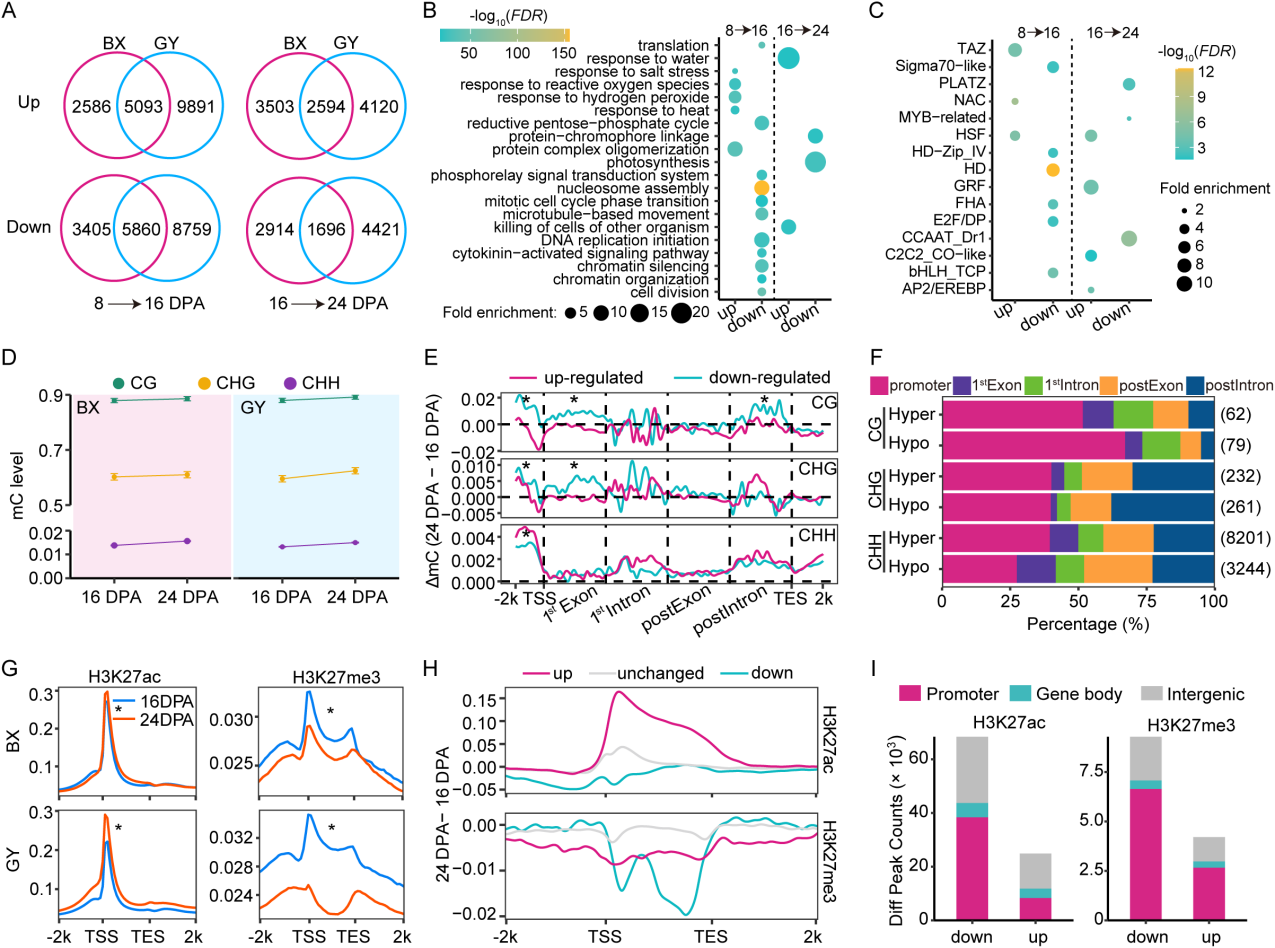
Transcription and epigenetic modification dynamics during grain development in the two experimental fields. **(A)** Differentially expressed genes from 8 to 16 DPA and from 16 to 24 DPA in BX and GY. **(B)** GO enrichment analysis for up- and down-regulated genes in both BX and GY from 8 to 16 DPA and from 16 to 24 DPA. **(C)** Enrichment analysis for transcription factors (TFs) among up- and down-regulated genes in both BX and GY from 8 to 16 DPA and from 16 to 24 DPA. **(D)** Comparison of global DNA methylation levels at CG, CHG, and CHH contexts between 16 and 24 DPA in the two fields. **(E)** DNA methylation changes of development-induced DEGs in both fields relative to the changes of all genes on gene bodies and flanking regions between 16 and 24 DPA in BX. Red line indicates up-regulated expression genes (FC ≥ 2, FDR < 0.05) and blue line indicates down-regulated expression genes (FC ≤ 0.5, FDR < 0.05). Statistical analysis was done based on paired Student’s *t*-test. *, *P* < 0.01. **(F)** Percentage of DMRs in different functional regions between 16 and 24 DPA in BX. The numbers in parentheses represent the total number of DMRs. **(G)** Comparison of H3K27ac and H3K27me3 levels across gene body and flanking regions between 16 and 24 DPA in the two fields. Statistical analysis was done based on paired Student’s *t*-test. *, *P* < 0.01. **(H)** The intensity variation of H3K27ac and H3K27me3 across gene body and flanking regions of development-induced genes between 16 and 24 DPA in BX. **(I)** Distribution of differentially modified peaks (DMPs) for H3K27ac and H3K27me3 between 16 and 24 DPA in BX and GY.

To investigate the effect of epigenetic regulation on gene expression during grain development, we identified DEGs between 8–16 DPA and 16–24 DPA in both BX and GY. In BX, 7,679 genes were up-regulated and 9,265 were down-regulated from 8–16 DPA, while GY exhibited more pronounced changes, with 14,984 genes up-regulated and 14,619 down-regulated during the same period (**Figure 2A**). From 16–24 DPA, BX showed 6,097 up-regulated and 4,610 down-regulated genes, compared to 6,714 up-regulated and 6,117 down-regulated genes in GY (**Figure 2A**). These results suggest that GY exerts a stronger overall impact on transcriptional remodeling during grain development. Genes exhibiting conserved expression changes across different environments during the same developmental period were classified as development-induced genes. We identified 10,953 such genes during 8–16 DPA and 4,290 during 16–24 DPA, indicating more active transcriptional reprogramming during early-to-mid stages of grain development (**Figure 2A**). Gene ontology (GO) analysis of these development-induced genes revealed significant functional divergence between genes that were expressed at different stages. From 8 to 16 DPA, upregulated genes were primarily involved in protein complex oligomerization and stress response (e.g., salt, heat and oxidative), while downregulated genes were associated with the reductive pentose-phosphate cycle, nucleosome assembly, and DNA replication initiation (**Figure 2B**). From 16 to 24 DPA, the genes related to water stress and other biology stress were upregulated, whereas the protein−chromophore linkage and photosynthesis related genes were downregulated (**Figure 2B**). The greater diversity of differentially expressed genes in early stages suggests increased complexity of gene regulatory networks during these stages. TF enrichment analysis of development-induced genes revealed stage-specific regulatory patterns (**Figure 2C**). From 8 to 16 DPA, TFs in the Sigma70-like, HD, bHLH_TCP, E2F/DP, FHA, and HD-Zip_IV families were significantly downregulated, while those in the TAZ, HSF, and NAC families were upregulated. Notably, HSF remained upregulated from 16 to 24 DPA, alongside three additional upregulated TFs (GRF, C2C2_CO-like, and AP2/EREBP). Conversely, CCAAT_Dr1, PLATZ, and MYB-related TFs were downregulated during this later stage (**Figure 2C**). These dynamic expression patterns demonstrate the distinct roles of different TF families in regulating grain development at different phases.

DNA methylation patterns dynamically changed during grain development. In BX, the bulk average DNA methylation levels in CG, CHG, and CHH contexts at 16 DPA were 87.9%, 60.2%, and 1.4%, respectively, increasing slightly to 88.6%, 61.0%, and 1.6% at 24 DPA (**Figure 2D**). Similar trends were observed in GY, with DNA methylation levels in CG, CHG, and CHH contexts rising from 87.9%, 59.5%, and 1.3% at 16 DPA to 89.2%, 62.4%, and 1.5% at 24 DPA (**Figure 2D**). These findings align with DNA methylation reprogramming observed in other plants, such as chickpea (Rajkumar et al., 2020) and Arabidopsis (Kawakatsu et al., 2017), underscoring the conserved role of DNA methylation in grain development. To further explore how DNA methylation regulates gene expression during this process, we investigated DNA methylation levels of development-induced genes in both experimental fields. For up-regulated genes, DNA methylation levels significantly decreased in the promoter and first exon regions in CG and CHG contexts. Conversely, down-regulated genes showed significant increases in methylation at these regions in both BX and GY (**Figure 2E**; **Supplementary Figure S3**), suggesting a strong association between DNA methylation dynamics and the transcriptional regulation of development-induced genes during grain development. We further identified DMRs during grain development. In BX, 581 CG-DMRs, 2,097 CHG-DMRs, and 100,260 CHH-DMRs were detected, whereas GY exhibited more DMRs, including 4,095 CG-DMRs, 17,978 CHG-DMRs, and 108,129 CHH-DMRs (**Supplementary Figure S4**). Notably, differential methylation was most prevalent in the CHH context in both BX and GY, with GY showing stronger CHG methylation changes than BX. Genomic distribution analysis revealed that 24.27% of CG-DMRs, 23.51% of CHG-DMRs, and 11.41% of CHH-DMRs in BX were located in promoter and gene body regions (**Figure 2F**). Similarly, in GY, 30.72% of CG-DMRs, 23.63% of CHG-DMRs, and 13.35% of CHH-DMRs overlapped with these functional regions (**Supplementary Figure S5**), suggesting their potential regulatory role in gene expression during grain development.

Histone modifications also exhibited dynamic changes during grain development. The average genic H3K27me3 level significantly decreased from 16 to 24 DPA, whereas H3K27ac showed the opposite trend, with higher levels detected at 24 DPA compared to 16 DPA in both BX and GY (**Figure 2G**). For development-induced genes, up-regulated genes showed significantly higher H3K27ac levels across the gene body, particularly around the transcriptional start site (TSS), whereas down-regulated genes displayed a marked reduction (**Figure 2H**; **Supplementary Figure S6**). Intriguingly, H3K27me3 levels decreased significantly for both up- and down-regulated genes from 16 to 24 DPA (**Figure 2H**; **Supplementary Figure S6**), suggesting distinct regulatory roles for these marks in transcriptional regulation. We further identified differentially modified peaks (DMPs) of histone modifications during grain development. In the two fields, H3K27ac-DMPs and H3K27me3-DMPs decreased from 16 to 24 DPA, with H3K27me3-DMPs showing more dramatic changes (**Supplementary Figure S7**). In both BX and GY, 24,915 H3K27ac-DMPs showed increased levels while 68,417 showed decreased levels at 24 DPA (**Figure 2I**). For H3K27me3, a total of 4,213 up-DMPs and 9,278 down-DMPs were detected at the same developmental stage in both BX and GY (**Figure 2I**). Approximately 49.93% of H3K27ac-DMPs and 23.52% of H3K27me3-DMPs overlapped with promoter and gene body regions (**Figure 2I**). These results indicated conserved epigenetic reprogramming and the important role of epigenetic regulation in reshaping gene expression during grain development.

### 3.4 Transcriptional and epigenetic responses to environmental differences

**Figure 3:**
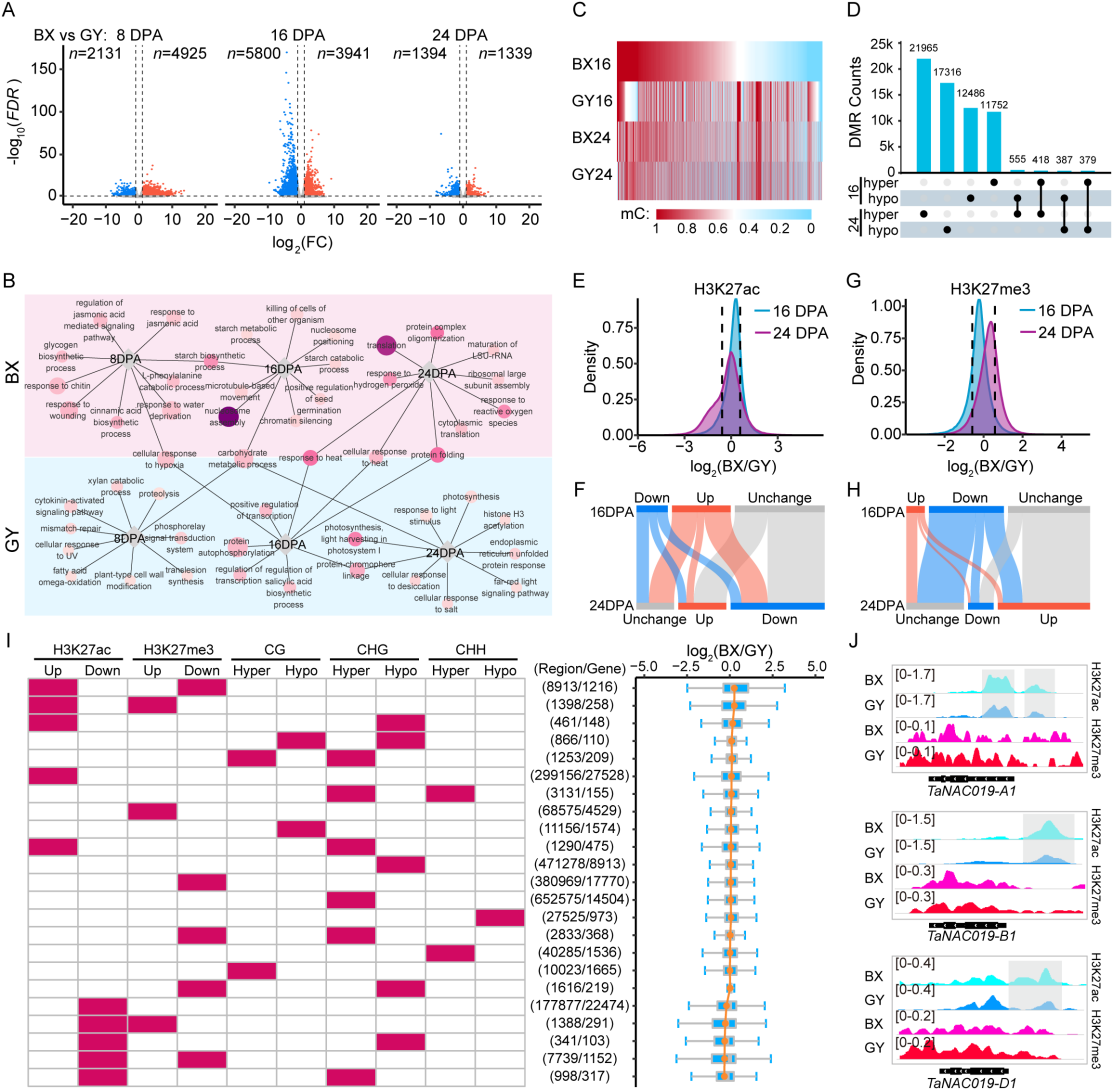
Transcriptional and epigenetic divergence during grain development between BX and GY. **(A)** Scatter plots of differential transcription levels in BX relative to GY at 8, 16, and 24 DPA against log_2_FC. Orange dots indicate up-regulated expression genes (FC ≥ 2, FDR < 0.05), blue dots indicate down-regulated expression genes (FC ≤ 0.5, FDR < 0.05). **(B)** GO enrichment analysis for the DEGs at 8,16, and 24 DPA between BX and GY. Circle size represents the number of genes, and the darker circle colors indicate higher statistical significance. **(C)** Identified CG-DMRs at 16 and 24 DPA between BX and GY. **(D)** Overlap of hypomethylated and hypermethylated CG-DMRs between BX and GY at 16 and 24 DPA. **(E)** Density plot displaying H3K27ac changes between BX and GY at 16 and 24 DPA. **(F)** Differentially modified peaks of H3K27ac between BX and GY at 16 and 24 DPA. **(G)** Density plot displaying H3K27me3 changes between BX and GY at 16 and 24 DPA. **(H)** Differentially modified peaks of H3K27me3 between BX and GY at 16 and 24 DPA. **(I)** Statistics of epigenetic modification differences between BX and GY across genomic regions at 16 DPA, along with expression changes of overlapping genes, sorted by fold change (log_2_(BX/GY)) from top to bottom. **(J)** Enriched signal of H3K27ac and H3K27me3 on three homoeologs of *TaNAC019* genes at 16 DPA in BX and GY. Gray box represents significantly higher peaks of H3K27ac in BX than in GY.

To dissect the impact of environmental factors on transcriptional and epigenetic regulation during grain development, we initially examined differential expression patterns between BX and GY at the same developmental stages. Specifically, compared with GY, BX exhibited 2,131 down-regulated and 4,925 up-regulated genes at 8 DPA. By 16 DPA, the number of DEGs increased substantially, with 5,800 down-regulated and 3,941 up-regulated genes (**Figure 3A**). At 24 DPA, the number of DEGs declined sharply, with only 1,394 down-regulated and 1,339 up-regulated genes remaining (**Figure 3A**). These results suggested that gene expression differences between the two locations are more dynamic during early-to-mid grain development stages. Then, we performed GO functional enrichment analysis to elucidate functional differences between stage-based DEGs in the two fields (**Figure 3B**). The analysis revealed that location-specific highly expressed genes were associated with distinct biological functions, with only a few shared categories between the two fields. Notably, these common genes displayed divergent temporal expression patterns between the two fields. For instance, carbohydrate metabolism-related genes were up-regulated at 16 DPA in BX, but highly expressed at 8 DPA and 24 DPA in GY. Similarly, heat response genes peaked at 16 DPA in GY but at 24 DPA in BX. This delayed expression in BX may be associated with the lag of a certain environmental factor. Meanwhile, starch biosynthetic genes were highly expressed at 8 DPA and 16 DPA in BX, with starch metabolic and catabolic process-related genes enriched at 16 DPA. In contrast, GY showed up-regulation of photosynthesis-related genes at 16 DPA and 24 DPA. These findings suggest that the two environments likely presented distinct environmental factors, driving differential gene expression patterns to optimize physiological and metabolic processes for local adaptation.

Significant DNA methylation changes were induced by distinct environmental conditions between the two experimental fields. We first detected DNA methylation changes in all protein-coding genes between BX and GY. At 16 DPA, CG methylation decreased significantly across promoter and gene body regions in BX, while CHG methylation increased notably in promoters (**Supplementary Figure S8**). By 24 DPA, both CG and CHG methylation decreased significantly in genic regions, particularly in promoter region in BX (**Supplementary Figure S7**). CHH methylation levels in BX were higher than in GY at both 16 and 24 DPA (**Supplementary Figures S8, S9**). Notably, distinct DNA methylation patterns were observed for DEGs between BX and GY. Although overall DNA methylation levels were significantly lower in BX than in GY, genes with higher expression in BX exhibited even lower DNA methylation levels in the CG context across promoters and gene bodies, along with lower CHG methylation in gene bodies at 16 DPA (**Supplementary Figure S9**). Conversely, these genes showed higher CHH methylation in gene bodies relative to GY at the same stage (**Supplementary Figure S9**). The opposite methylation pattern was observed for lower expression genes in BX (**Supplementary Figure S9**). We next identified DMRs resulting from environmental differences between the two fields. At 16 DPA, we detected 1,276,896 DMRs between BX and GY, consisting of 12,549 (0.98%, CG), 679,736 (53.23%, CHG), and 47,749 (3.74%, CHH) hypomethylated regions, compared to 13,428 (1.05%, CG), 490,586 (38.42%, CHG), and 32,848 (2.57%, CHH) hypermethylated regions in GY (**Figures 3C, D**; **Supplementary Figures S10A, S11A**). By 24 DPA, the total number of DMRs between BX and GY decreased to 1,156,201, with 22,938 (1.98%, CG), 430,378 (37.22%, CHG), and 42,656 (3.69%, CHH) showing hypomethylation, while 18,082 (1.56%, CG), 597,257 (51.66%, CHG), and 44,890 (3.88%, CHH) exhibited hypermethylation in GY (**Figures 3C, D**; **Supplementary Figures S10A, S11A**). Only a small proportion of DMRs maintained consistent methylation patterns between BX and GY at 16 and 24 DPA, including 805 (1.23%) CG-DMRs, 59,333 (2.86%) CHG-DMRs, and 2,334 (1.43%) CHH-DMRs. In contrast, 934 (1.43%) CG-DMRs, 63,906 (3.08%) CHG-DMRs, and 2,419 (1.48%) CHH-DMRs displayed opposite methylation patterns between these two stages (**Figure 3D**; **Supplementary Figures S10B, S11B**). The stage-specific nature of most DMRs suggests that environmental effects on genomic DNA methylation dynamically change during grain development.

Dramatic alterations in histone modifications were observed between BX and GY at both 16 and 24 DPA. At 16 DPA, BX exhibited higher H3K27ac but lower H3K27me3 levels compared with GY (**Figures 3E, G**). In contrast, at 24 DPA, BX showed lower H3K27ac and higher H3K27me3 levels than GY (**Figures 3E, G**). Promoter and TSS-proximal regions showed the greatest sensitivity to H3K27ac changes induced by environmental variation, with higher levels at 16 DPA but lower levels at 24 DPA in BX compared with GY (**Supplementary Figure S12**). However, gene bodies displayed the most pronounced differences in H3K27me3, with BX having significantly lower levels than GY at 16 DPA but higher levels at 24 DPA (**Supplementary Figure S12**). We further identified DMPs between BX and GY during grain development. At 16 DPA, 123,576 peaks exhibited significantly higher H3K27ac levels in BX compared with GY, while 66,128 peaks showed lower levels. By 24 DPA, this pattern reversed, with 101,334 peaks displaying higher H3K27ac levels and 198,443 peaks showing lower levels in BX relative to GY (**Figure 3F**). Notably, only 12.31% (15,214) of the higher H3K27ac peaks in BX and 34.19% (22,612) of the higher peaks in GY at 16 DPA retained the same differential pattern at 24 DPA (**Figure 3F**). For H3K27me3, at 16 DPA, 23,457 peaks had higher levels in BX compared with GY, while 95,039 peaks showed the opposite trend (**Figure 3H**). At 24 DPA, 101,334 peaks were enriched in BX, while only 32,382 peaks were enriched in GY (**Figure 3H**). Among these DPMs, only 27.16% (6,372) of the higher H3K27me3 peaks in BX and 11.75% (11,166) of the higher peaks in GY at 16 DPA retained the same differential pattern at 24 DPA.

To investigate whether environmentally induced changes in epigenetic modifications combinatorially regulate gene expression, we first identified all epigenetic modification changes at the same genomic region. Results showed that among all genomic regions exhibiting epigenetic modification changes, 97.99% and 97.29% displayed alterations in only a single modification type between BX and GY at 16 and 24 DPA, respectively. However, further investigation revealed that co-occurring epigenetic modification changes on genes had greater regulatory effects on gene expression. For example, at 16 DPA, 8,913 regions in BX exhibited both higher H3K27ac levels and lower H3K27me3 levels compared with GY. These regions overlapped with 1,216 genes, which showed the greatest expression bias toward BX (**Figure 3I**). We further observed that genes in BX with elevated H3K27ac coupled with reduced CG/CHG methylation had relatively higher expression compared with GY (**Figure 3I**; **Supplementary Figure S13**). Conversely, genes exhibiting decreased H3K27ac, increased H3K27me3, and higher methylation levels in BX displayed correspondingly lower expression relative to GY (**Figure 3I**; **Supplementary Figure S13**). For example, at 16 DPA, *TaNAC019* showed significantly higher expression levels in BX compared with GY across all three subgenomes (**Supplementary Figure S14**). This activation was associated with elevated H3K27ac levels in the promoter regions of all *TaNAC019* homoeologs, along with reduced H3K27me3 levels specifically across the *TaNAC019-D1* promoter and gene body (**Figure 3J**).

Overall, environmental differences triggered extensive epigenetic changes, with BX exhibiting higher H3K27ac, lower H3K27me3, and reduced DNA methylation levels. These modifications collectively shaped expression divergence between BX and GY.

### 3.5 Underlying epigenetic basis for grain quality divergence between the two experimental fields

**Figure 4:**
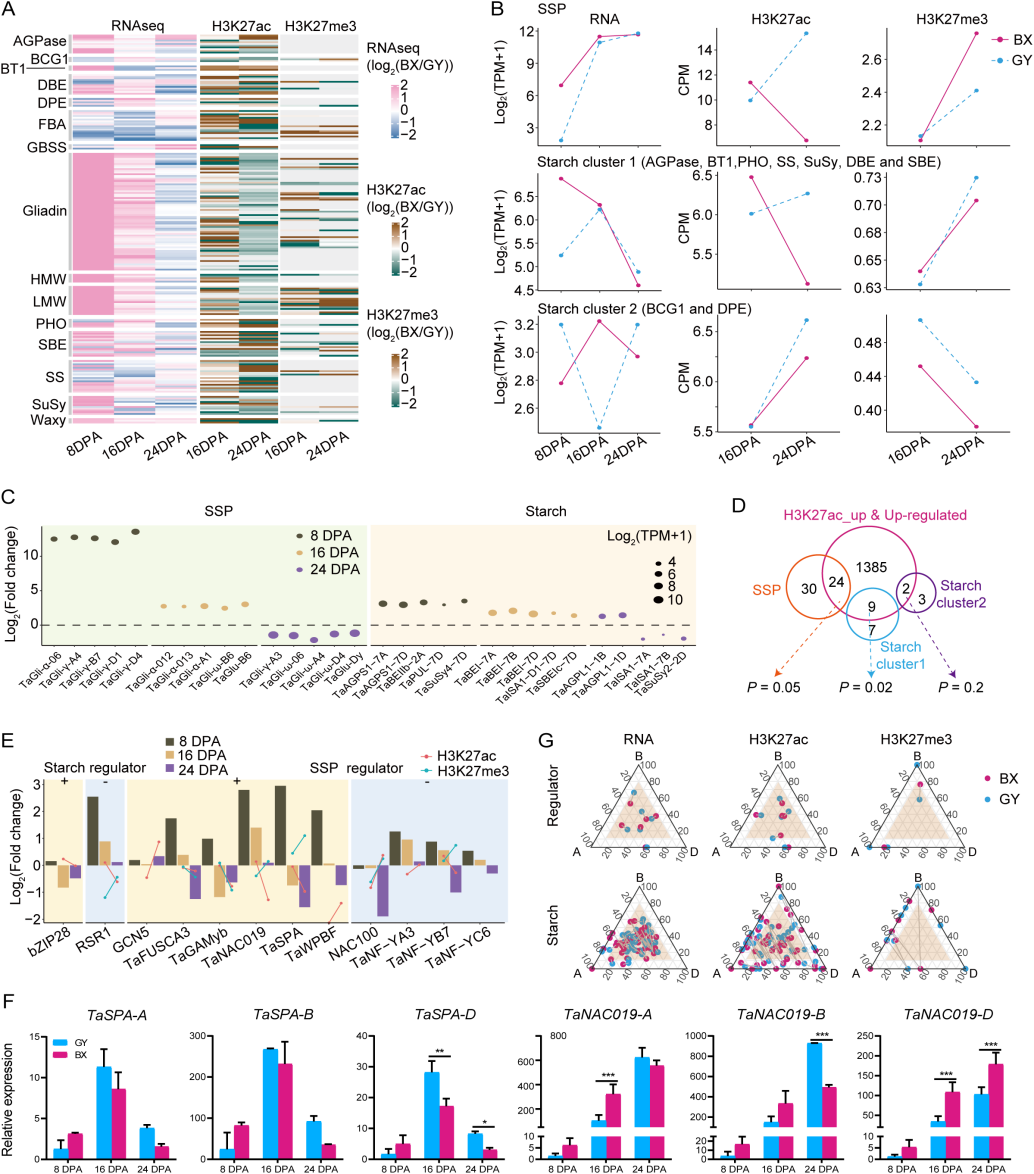
Epigenetic regulation of starch and SSP coding genes between BX and GY during grain development. **(A)** The comparison of gene expression (left) and histone modification intensity (right) of SSP and starch encoded genes between BX and GY at 16 and 24 DPA. **(B)** The key starch and SSP synthesis genes with the most significant expression differences between BX and GY across different developmental stages. **(C)** The expression level and modification level of H3K27ac and H3K27me3 on the three groups of SSP and starch encoded genes in BX and GY. **(D)** Overlap between higher expressed starch and SSP synthesis genes and genes with increased H3K27ac and expression levels in BX at 16 DPA. **(E)** Variation in expression, H3K27ac and H3K27me3 of key regulators for starch and SSP synthesis in BX versus GY at different developmental stages. **(F)** The relative expression level of *TaSPA* and *TaNAC019* in ‘Fengdecunmai 5’ grains at 8, 16, and 24 DPA between BX and GY. qRT-PCR data were normalized to *TaActin*. Statistical significance was determined by two-way ANOVA. *, *P* ≤ 0.05; **, *P* ≤ 0.01; ***, *P* ≤ 0.001. **(G)** Ternary plot showing relative expression and epigenetics modification (H3K27ac and H3K27me3) abundance of regulators and starch biosynthesis genes at 16 DPA. Each circle represents a gene triad with A, B, and D coordinates consisting of the relative contribution of each homoeolog to the overall triad. The dash between each circle represents the same gene triad in BX and GY. Balanced triads are shown within a brown shade.

Considering the significant divergence in grain quality between BX and GY (**Figures 1A, B**), we further investigated the effects of epigenetic modifications on the expression of starch- and SSP-related genes in the two experimental fields. We first conducted a detailed analysis of SSP-coding genes (HMW-GS, LMW-GS, and gliadin) and major starch synthesis genes, including ADP-glucose pyrophosphorylases (*AGPases*), granule-bound starch synthases (*GBSSs*), starch synthases (*SSs*), starch branching enzymes (*SBEs*), debranching enzymes (*DBEs*), starch/α-glucan phosphorylases (*PHOs*), BRITTLE1 (*BT1*), disproportionating enzymes (*DPEs*), branching enzyme gene 1 (*BCG1*), sucrose synthase (*SuSy*), fructose-1,6-bisphosphate aldolase (*FBA*), and *Waxy*, examining their expression patterns and epigenetic modifications during endosperm development in both fields (**Supplementary Table S3**). Interestingly, among the 100 analyzed SSP genes, all showed higher expression in BX than in GY at 8 DPA, with 97 being significantly upregulated (**Figure 4A**). At 16 DPA, 94 SSPs maintained higher expression in BX, including 54 that were significantly more highly expressed. By 24 DPA, however, the trend reversed, with 78 SSPs exhibiting higher expression in GY, 13 of which were significant. A similar pattern was observed for starch-related genes. At 8 DPA, 58 of the 87 analyzed starch-related genes showed higher expression levels in BX, with 50 being significantly upregulation (**Figure 4A**). While 56 genes still favored BX at 16 DPA, only 16 remained significantly higher. By 24 DPA, expression reversed toward GY, with 51 genes more highly expressed in GY, including 13 that were significantly elevated (**Figure 4A**).

To elucidate the substantial differences in starch and protein traits observed between the two environmental fields, we explored the key starch and SSP synthesis genes showing the most significant expression differences across the two environments. SSP genes exhibited more dynamic changes with higher fold-changes than starch genes (**Figure 4B**). Notably, *γ-gliadins*, *α-gliadins*, *ω-gliadins*, and the LMW glutenin gene *TaGlu-B6* showed significantly higher expression in BX at 8 and/or 16 DPA, while some *ω-gliadins* and the HMW glutenin gene *TaGlu-Dy* were downregulated in BX at 24 DPA, but with lower fold-changes. These expression patterns likely explain the significantly higher accumulation of LMW-GS, α/β-gliadin, and γ-gliadin in grains under BX conditions compared with those under GY (**Figure 1B**). Among starch synthesis genes, *AGPases*, *SuSy4*, pullulanase (*TaPUL*), *TaBEI*, and *TaSBE* were upregulated in BX compared with GY at all three stages, except *SuSy2* and the amylopectin synthesis gene *Triticum aestivum* isoamylase 1 (*TaISA1*). This consistent upregulation may contribute to BX’s higher amylose content relative to GY (**Figure 1B**).

The transcriptional dynamics and epigenetic modification changes showed strong correlations for both SSPs and starch synthesis genes. SSP genes maintained high expression levels from 8 to 16 DPA before declining at 24 DPA in BX compared with GY. Corresponding changes in H3K27ac and H3K27me3 were observed between BX and GY from 16 to 24 DPA. At 16 DPA, BX exhibited higher H3K27ac and lower H3K27me3 levels than GY, while the opposite pattern occurred at 24 DPA (**Figures 4A, C**). Starch biosynthesis genes displayed higher complexity, with their expression patterns being categorized into two distinct clusters (**Figure 4C**). Starch cluster 1 genes showed significantly higher expression in BX at 8 DPA, followed by increased expression in GY and decreased expression in BX, which resulted in similar levels at 16 and 24 DPA in both environments (**Figure 4C**). In contrast, starch cluster 2 genes exhibited opposite expression patterns at all three stages between the two fields, though H3K27ac and H3K27me3 levels followed the same trends at 16 and 24 DPA in both environments (**Figure 4C**). Given the substantial epigenetic effects on starch and SSP biosynthesis gene expression (**Figures 4A, C**), we analyzed correlations between expression changes and epigenetic modifications. Significantly increased expression of starch (*P* = 0.02, hypergeometric test) and SSP (*P* = 0.05, hypergeometric test) genes correlated with higher H3K27ac levels in BX versus GY, with 44% of upregulated SSP genes and 56% of starch cluster 1 genes in BX showing significantly higher H3K27ac peaks (**Figure 4D**). However, the correlations between expression changes of these genes and alterations in H3K27me3 or DNA methylation were markedly weaker than those observed for H3K27ac.

We further analyzed expression changes of regulators directly controlling starch- and/or SSP-related genes (**Figure 4E**). For starch biosynthesis, the positive regulator *bZIP28* (Song et al., 2020) showed lower expression in BX than GY at 16 and 24 DPA, despite higher H3K27ac levels in BX at 16 DPA. The negative regulator *RSR1* (Liu et al., 2016) was upregulated throughout grain development in BX but with diminishing magnitude, potentially explained by corresponding H3K27ac and H3K27me3 changes. Among SSP regulators, the positive regulator *TaNAC019* (Gao et al., 2021) displayed similar expression and modification level changes as *RSR1*. *GCN5*, which positively regulates SSP synthesis (Guo et al., 2015; Zhang et al., 2024b), was up-regulated at all stages, particularly pronounced at 8 and 16 DPA (**Figure 4F**), likely attributable to significantly elevated H3K27ac during this period. Meanwhile, another positive regulator *TaSPA* (Boudet et al., 2019) was upregulated at 8 DPA but declined sharply to levels below GY by 16 and 24 DPA (**Figure 4F**), potentially induced by concurrent H3K27ac downregulation and H3K27me3 upregulation in BX. The negative regulator *TaNF-YB7* (Chen et al., 2024) displayed higher expression in BX at 8 and 16 DPA but decreased at 24 DPA to the level below that in GY, which may be caused by the similar histone modification as that of *TaSPA*. Starch and regulator expression showed similar subgenome-biased patterns in both environments, while epigenetic modifications exhibited greater BX-GY differentiation, highlighting environmental influences on epigenetic regulation (**Figure 4G**; **Supplementary Figure S15**).

In conclusion, the observed divergence in grain quality between BX and GY was primarily driven by differential expression of SSPs, starch synthesis genes, and their transcriptional regulators, and environmentally induced epigenetic modifications significantly modulated the expression patterns of these genes, ultimately contributing to phenotypic variation.

### 3.6 Unraveling the transcriptional networks underlying grain quality divergence between the two experimental fields

**Figure 5:**
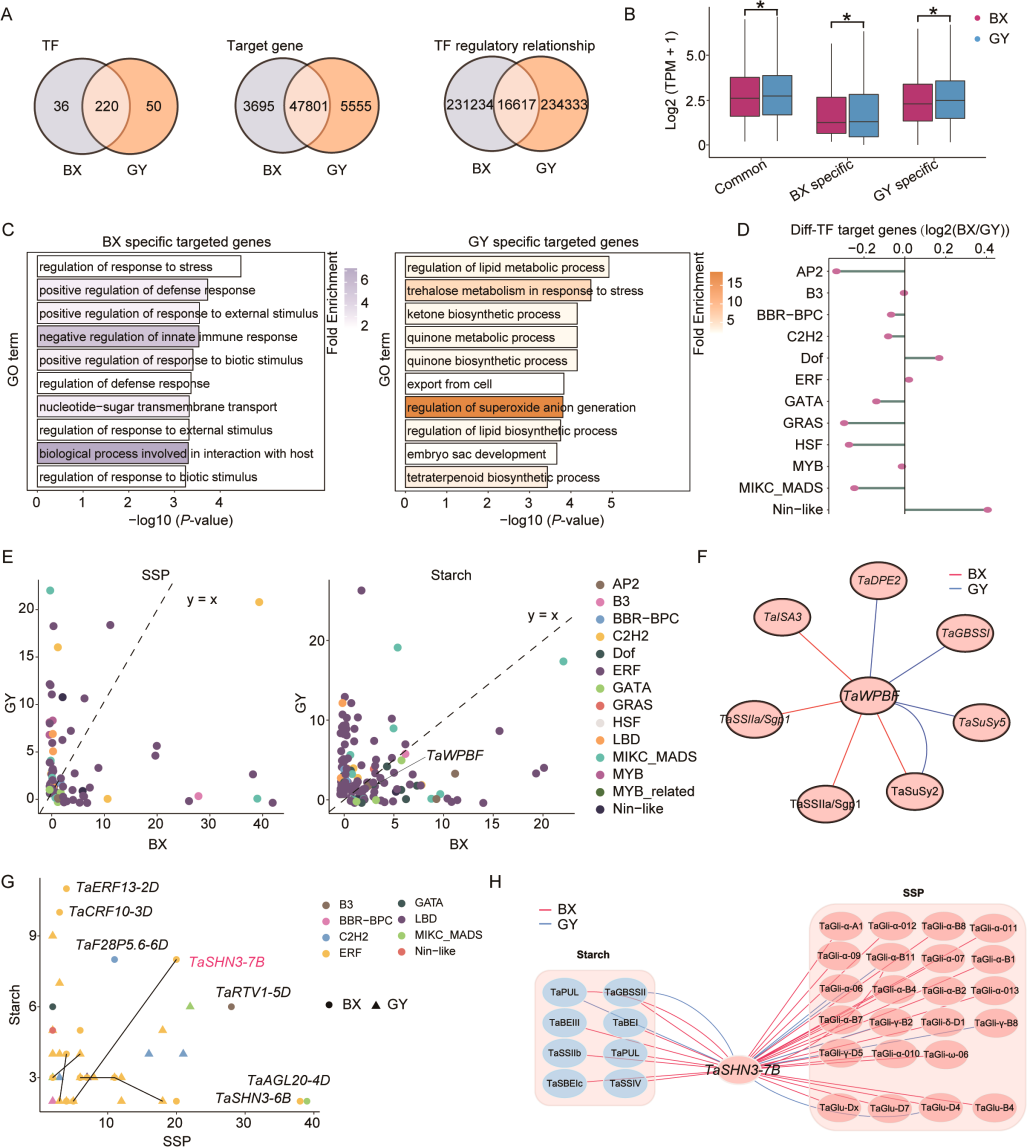
Comparison of transcriptional regulatory networks between BX and GY. **(A)** Characteristic of TFs, target genes, and connections between BX and GY during grain development. **(B)** Comparison of gene expression levels among BX-specific, GY-specific and common genes between BX and GY. **(C)** GO enrichment analysis for BX-specific and GY-specific genes. **(D)** Expression differences of genes targeted by different TFs in BX versus GY. **(E)** Counts of SSPs- (left) and starch-related genes (right) regulated by different TFs in BX and GY. **(F)** Differential regulatory network of *TaWPBF* in BX and GY. **(G)** Identification of regulators associated with both SSPs- and starch-related genes in BX and GY. **(H)** Differential regulatory network of *TaSHN3-7B* for SSP and starch between BX and GY.

To comprehensively decipher the intricate regulatory mechanisms underlying the divergence in starch biosynthesis and SSP accumulation between BX and GY during grain development, we constructed transcriptional regulatory networks (TRNs) for both BX and GY using gene co-expression data. The analysis revealed that the TRN for BX contained 51,752 nodes and 247,851 connections, whereas the TRN for GY comprised 53,626 nodes and 250,950 connections (**Figure 5A**). Based on the TRNs of BX and GY, we identified 220 TFs with conserved regulatory roles during grain development in both BX and GY, while 36 and 50 TFs were uniquely essential for BX and GY, respectively (**Figure 5A**). Among target genes, 47,801 were co-regulated in both BX and GY, with 3,695 and 5,555 genes specifically regulated in BX and GY, respectively. Although most genes were shared in both BX and GY, their regulatory relationships exhibited significant divergence (**Figure 5A**). This environmental-specific regulation likely contributed to the divergence of grain quality between BX and GY. The expression pattern of co-regulated genes in both BX and GY exhibited higher expression levels than BX- and GY-specific genes, indicating their core role in grain development regardless of environmental influences (**Figure 5B**). Notably, BX-specific genes showed significantly elevated expression in BX, while GY-specific genes were preferentially expressed in GY (**Figure 5B**). GO enrichment analysis revealed that BX-specific regulated genes were predominantly associated with environmental response, whereas GY-specific genes were enriched in biosynthetic and metabolic processes (**Figure 5C**). Further analysis revealed that genes targeted by Nin-like and Dof family TFs preferentially activated in BX, whereas those regulated by AP2, GRAS, HSF, and MIKC_MADS TFs showed greater activation in GY (**Figure 5D**), suggesting a key role for these TFs in mediating divergent grain development between the two fields.

The regulatory relationships between TFs and genes related to SSPs and starch were further investigated in both BX and GY. Results revealed that SSP expression was associated with 42 TFs in BX compared to 65 TFs in GY (**Figure 5E**). A similar trend was observed for starch-related genes, with 88 TFs playing key regulatory roles in BX versus 124 TFs in GY. Although fewer TFs regulated SSPs in BX, more TF-SSP interactions were detected there, whereas GY exhibited more connections for starch-related genes. Among these key regulators, 21 TFs influenced SSPs and 63 TFs modulated starch-related genes in both environments, though their specific regulatory relationships diverged significantly between the two environments. For instance, in BX, *TaWPBF* regulated the starch-related genes *TaISA3*, *TaSSIIa*, and *TaSuSy2*, whereas in GY, *TaWPBF* was more strongly associated with *TaDPE2*, *TaGBSSI*, *TaSuSy2*, and *TaSuSy5* (**Figure 5F**). Furthermore, we identified 29 TFs (primarily from the ERF family) potentially regulating both SSPs and starch-related genes (**Figure 5G**). However, substantial differences in regulatory relationships were observed between BX and GY. For instance, despite the minimal difference in gene expression, *TaSHN3-7B* regulated 19 SSPs and 7 starch-related genes in BX, but retained connections to only 4 SSPs and 2 starch-related genes in GY (**Figure 5H**; **Supplementary Figure S16**). These findings demonstrate a comprehensive restructuring of TRNs underlying grain quality divergence between BX and GY.

## 4 Discussion

The quality traits of wheat cv. ‘Fengdecunmai 5’ exhibit significant variation under different environmental conditions. Given the established roles of DNA methylation (Zhou et al., 2022) and histone modifications such as H3K27ac and H3K27me3 (Chen et al., 2024; He et al., 2024; Liu et al., 2025; Zhao et al., 2024) in wheat endosperm development and starch/SSP synthesis, we hypothesized that these epigenetic modifications contribute to the observed quality trait variations in ‘Fengdecunmai 5’ across environments. This study systematically investigated the impact of DNA methylation and H3K27ac/H3K27me3 modifications on starch and SSP synthesis in response to different environmental conditions using wheat cv. ‘Fengdecunmai 5’ at developmental stages 8, 16 and 24 DPA grown in BX and GY. We found that ‘Fengdecunmai 5’ sown in GY exhibited more dynamic gene expression changes during seed development. Notably, combined epigenetic modifications exerted stronger regulatory effects on gene expression than individual modification types. Among these modifications, variation in histone modifications (particularly H3K27ac, **Figure 4D**) showed a stronger correlation with starch and SSP differences between the two fields than DNA methylation did. Furthermore, TRN analysis identified 29 candidate TFs with potential dual regulation of both SSP and starch-related genes. Among them, *TaSHN3-7B* emerged as a key predicted regulator accounting for the substantial differences in starch and SSP content between BX and GY.

In the present study, we observed a progressive decrease in both expressed genes (**Figure 1D**) and development-induced genes (**Figure 2A**) during seed development in both experimental fields, suggesting more pronounced transcriptional reprogramming during early-to-mid grain development stages. This expression pattern aligns with expression trends reported in previous studies (Chi et al., 2019; Shewry et al., 2012). As the number of expressed genes decreased, DNA methylation levels increased slightly in both fields as seeds developed (**Figure 2D**), consistent with methylation reprogramming patterns observed in *Arabidopsis* (Kawakatsu et al., 2017), chickpea (Rajkumar et al., 2020), and soybean (An et al., 2017; Lin et al., 2017). Meanwhile, histone modifications also exhibited dynamic changes during grain development, with average genic H3K27me3 levels decreasing while H3K27ac levels increased significantly in both BX and GY (**Figure 2G**). Notably, we detected an unexpected moderate positive correlation between H3K27ac and H3K27me3 densities (r = 0.36~0.59) (**Figure 1E**). Genomic regions co-marked by both activating and repressive histone modifications are known as bivalent chromatin domains, which can establish poised transcriptional states for precise spatiotemporal regulation. Previous studies have identified various bivalent chromatin states in plants. In pea (*Pisum sativum L.*), the H3K9ac-H3K27me3 bivalent chromatin state has been shown to regulate developmental processes and stress responses (Wan et al., 2024). Similarly, *Brassica napus* possesses an H3K4me1-H3K27me3 bivalent chromatin state that modulates tissue-specific gene expression (Zhang et al., 2021). Moreover, H3K27me3-H3K18ac bivalent chromatin plays an important role in responding to pathogen signals in *Arabidopsis* (Zhao et al., 2021). However, the biological implications of the co-occurring H3K27ac and H3K27me3 modifications observed in our study require further experimental validation.

During early grain development stages, gene regulatory networks exhibit greater complexity, as evidenced by the increased diversity of development-induced genes (**Figure 2B**). For genes identified at 16 and 24 DPA, up-regulated genes showed significantly lower DNA methylation levels in CG and CHG contexts coupled with higher H3K27ac levels across gene bodies, while down-regulated genes displayed a marked reduction in H3K27ac accompanied by increased DNA methylation levels (**Figures 2E, H**; **Supplementary Figures S3, S6**). Intriguingly, H3K27me3 levels decreased significantly for both up- and down-regulated genes from 16 to 24 DPA (**Figure 2H**; **Supplementary Figure S6**). This observed pattern aligns with findings from another study (Zhao et al., 2023), suggesting H3K27me3 may play a limited role in direct transcriptional regulation during these developmental stages.

The DEGs between BX and GY were enriched at 8 and 16 DPA, revealing active dynamic differences in gene expression during the early-to-mid grain development stages. As expected, the highly expressed genes in the two environments were involved in specialized physiological processes (**Figure 3B**), indicating that the differential gene expression patterns were triggered by distinct environmental factors between BX and GY. Although there were shared categories between the two fields, different temporal expression patterns were observed, which was likely caused by the lag of certain environmental factors between the two environments. At the same time, the above-mentioned modifications collectively shaped the expression divergence between these two fields. In most cases, the regulatory effect of a single type of modification was not greater than that of co-occurring modification changes. The genes that were specifically highly expressed under particular environmental conditions had elevated H3K27ac coupled with reduced CG/CHG methylation or H3K27me3 in both locations, as exemplified by the correlation between elevated *TaNAC019* expression levels and correspondingly increased H3K27ac coupled with reduced H3K27me3 levels in BX (**Figure 3J**). Additionally, the low percentage of shared DMRs or DMPs between 16 and 24 DPA in BX and GY (**Figures 3D, F, H**; **Supplementary Figures S10, S11**) revealed the stage-specific properties of epigenetic modifications.

In general, ‘Fengdecunmai 5’ planted in BX had a higher amylose ratio and higher gliadin and glutenin contents but lower starch content across nearly all developmental stages compared with GY (**Figure 1B**). Indeed, the expression levels of some key genes involved in SSP accumulation and/or starch biosynthesis during grain development, along with their corresponding histone modifications, differed significantly between the two environments. Similar to the aforementioned *TaNAC019*, almost all analyzed SSP genes exhibited higher expression and H3K27ac levels in BX at early-to-mid stages (**Figure 3A**), particularly *γ-gliadins*, *α-gliadins*, *ω-gliadins*, and the LMW glutenin gene *TaGlu-B6* (**Figure 3B**), consistent with the “stage-specific transcriptional divergence” model. A similar pattern was observed for starch-related genes: more than half showed higher expression and H3K27ac levels in BX at 8 and 16 DPA (**Figure 3A**). Starch biosynthesis involves two sequential steps: first, photosynthetic products and sucrose are hydrolyzed into glucose-1-phosphate (G1P), followed by enzymatic conversion of G1P into amylose and amylopectin (Ma et al., 2014). Accordingly, genes related to G1P generation, such as *Susy* and *FBA*, were predominantly expressed at the early stage (4 and 7 DPA), while amylose and amylopectin biosynthesis genes (e.g., *AGPase*, *DBE*, *GBSS*, *SBE, SS* and *waxy*) dominated at later stages (14 and 18 DPA) (He et al., 2024). Although starch-related genes generally showed higher expression levels in BX, most *FBA* genes were highly expressed at 24 DPA, which should peak at 8 DPA, and the majority of *AGPase*, *DBE*, *SS* genes dominated at an earlier phase (8 DPA), which should be highly expressed at 16 DPA (**Figure 3A**). Meanwhile, *NAC019-A1*, a reported negative regulator of starch synthesis in wheat developing endosperm (Liu et al., 2020), was highly expressed at 16 DPA in BX, with the most pronounced levels among all three *TaNAC019* homoeologs (**Supplementary Figure S14**). Another TF *RSR1*, which negatively regulates multiple starch synthesis-related enzyme genes (Liu et al., 2016), displayed the same trend as *NAC019-A1*, showing elevated expression in BX at 16 DPA (**Figure 4E, G**). The lower starch content in BX, despite the higher expression of starch biosynthesis genes, likely resulted from the disrupted temporal expression patterns of these genes and the elevated expression of *TaNAC019* and *TaRSR1.* Moreover, the higher amylose content at 16 and 24 DPA in BX may be attributed to the increased expression of photosynthesis-related genes (**Figure 3B**), *waxy* and a portion of *DBE* genes (**Figure 3A**) at these stages.

The correlation between SSP accumulation and/or starch biosynthesis gene expression and their corresponding histone modifications (**Figures 4C, D**) strongly suggests that, compared with H3K27me3 and DNA methylation, H3K27ac serves as the dominant epigenetic modification regulating the expression variation of these genes. Genome-wide studies in other crop species have elucidated the role of DNA methylation in seed development, including its involvement in starch biosynthesis in maize (Hu et al., 2021) and SSP accumulation and starch biosynthesis in rice (Zemach et al., 2010). Although DNA methylation has been reported to contribute to wheat development, such studies were limited to specific genes such as *TaGli-γ-2.1* (Zhou et al., 2022) and *HMW-GSs* (Zhang et al., 2018) rather than genome-wide analyses. The minimal role of DNA methylation changes in the expression variation of genes involved in SSP accumulation and starch biosynthesis observed here may reflect the fact that its function and status in these processes are more stable and conserved than those of H3K27ac.

On one hand, the high expression of 220 TFs and 47,801 target genes identified in the constructed TRN for ‘Fengdecunmai 5’ in both BX and GY (**Figure 5A**) revealed the conserved regulatory roles of these genes. On the other hand, the enriched environmental-specific genes associated with diverse biological functions (**Figures 5B, C**) implied significant differences in environmental factors between the two fields. Interestingly, we discovered that *TaWPBF* targets distinct starch synthesis genes in the two fields (BX: *TaISA3*/*TaSSIIa*; GY: *TaDPE2*/*TaGBSSI*), suggesting that this TF influences grain traits through environment-dependent regulatory mechanisms. Additionally, the number of TF target genes varied significantly between environments. For example, *TaSHN3-7B*, a newly identified TF in this study, extensively regulated SSPs in BX but exhibited reduced regulatory activity in GY, demonstrating its functional plasticity (**Figure 5H**). The greater number of *TaSHN3-7B* regulated genes in BX may account for the higher SSP content observed there (**Figure 1B**). Given the large number of SSP genes regulated by *TaSHN3-7B*, genome editing of this single TF might be more effective than targeting individual SSP genes, which could be done after the conserved function of the *TaSHN3-7B* has been confirmed.

Considering global climate change, ensuring stable crop quality remains a major challenge. Traditional breeding programs have largely focused on genetics, often overlooking environmental and epigenetic influences on phenotypic variability. In recent years, the development of molecular techniques such as genome, transcriptome, and epigenome sequencing has facilitated epigenome editing-based crop improvement, which relies on the identification of specific targets and stable, heritable epigenetic marks that are linked to desirable agronomic traits (Crisp et al., 2022; Rajpal et al., 2022; Turcotte et al., 2022). While numerous studies have examined wheat quality variations across environments, none have addressed this issue from an epigenetic perspective. The present study fills this critical gap by revealing the role of H3K27ac in regulating SSP- and starch-related genes and the corresponding quality differences between BX and GY. Importantly, we identified TaSHN3-7B as a key regulator of SSP accumulation. However, future investigations should (1) expand the scope to diverse wheat germplasms and environmental conditions, (2) elucidate the molecular crosstalk between specific histone marks (e.g., H3K27ac) and the TF like *TaSHN3-7B*, and (3) develop field-applicable epigenetic markers for breeding climate-resilient wheat with stable grain quality. Moreover, the development of cost-effective, high-throughput epigenome profiling platforms is urgently needed to facilitate the application of epigenetic regulatory mechanisms in practical wheat breeding programs, ultimately enabling precision improvement of complex agronomic traits.

## Data Availability

The datasets presented in this study can be found in online repositories. The names of the repository/repositories and accession number(s) can be found below: https://www.ncbi.nlm.nih.gov/, PRJNA1280545.
