# Supplementary material for: The dynamic epigenetic atlas and its effects on instability of starch and seed storage protein quality traits in wheat (Triticum aestivum L.)
Source: Front Plant Sci. 2025 Oct 24;16:1685120. doi: 10.3389/fpls.2025.1685120 (PMC12592202; doi:10.3389/fpls.2025.1685120)
Supplement: Supplementary file 1 [file DataSheet1.docx]

Supplementary Material

# Supplementary Data

Supplementary Material should be uploaded separately on submission. Please include any supplementary data, figures and/or tables.

Supplementary material is not typeset so please ensure that all information is clearly presented, the appropriate caption is included in the file and not in the manuscript, and that the style conforms to the rest of the article.

# Supplementary Figures and Tables

**
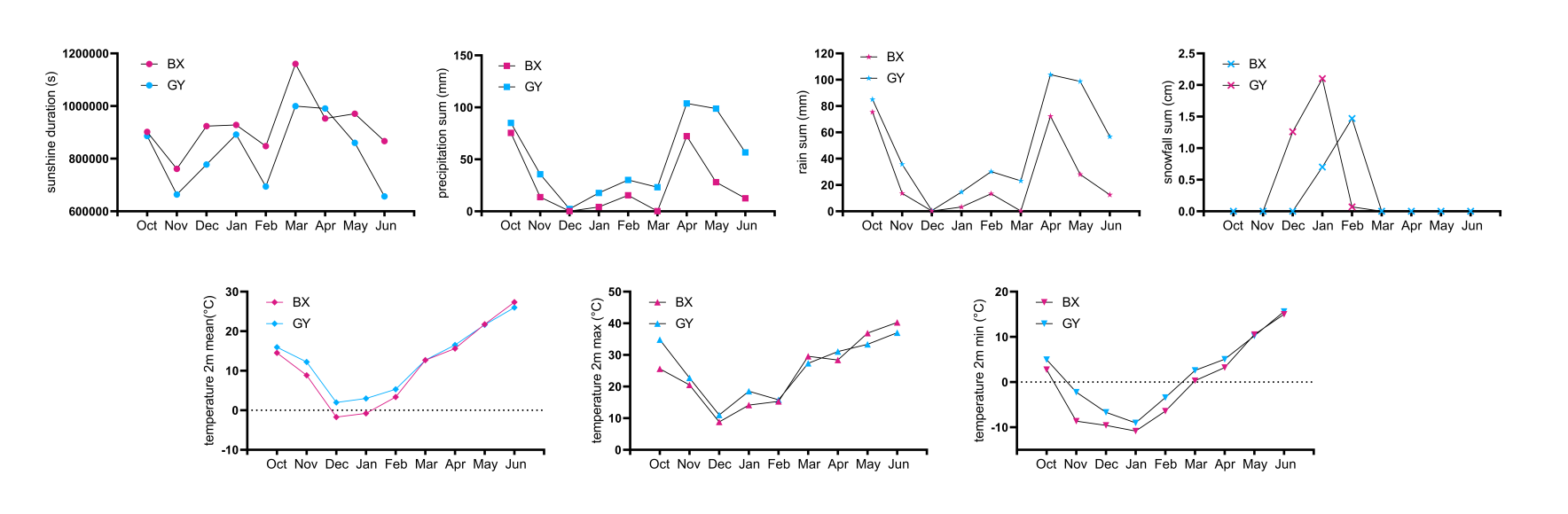
**For more information on Supplementary Material and for details on the different file types accepted, please see [here](https://www.frontiersin.org/guidelines/author-guidelines#supplementary-material).Supplementary Figures

**Supplementary Figure 1.** Meteorological Elements (Sunshine Duration, Precipitation Sum, Rain Sum, Snowfall Sum, and Temperature 2m Mean, Max and Min) at BX and GY (October 2022 to June 2023).

**
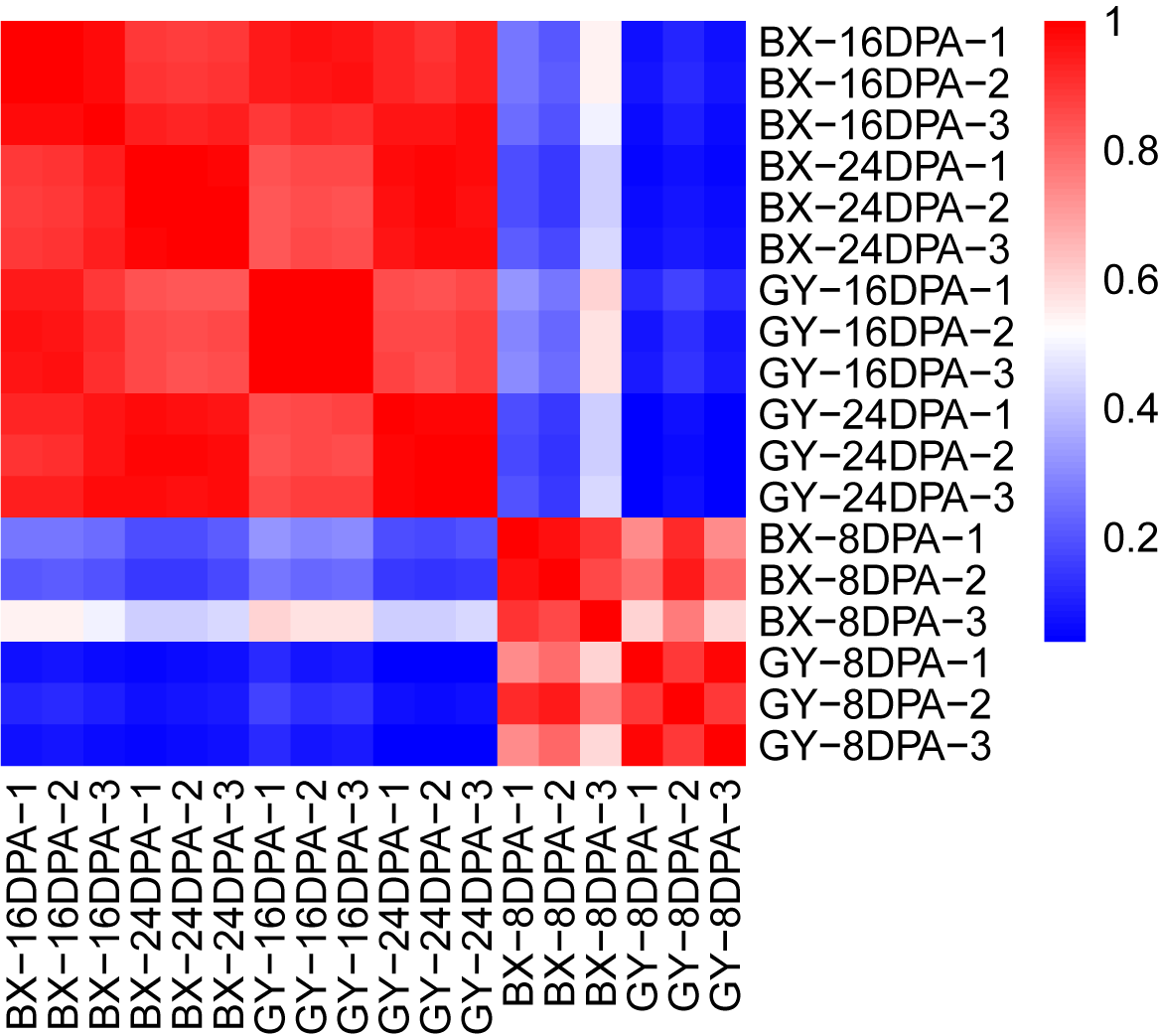
**

**Supplementary Figure 2.** RNA-Seq data correlation heatmap across all samples.

**
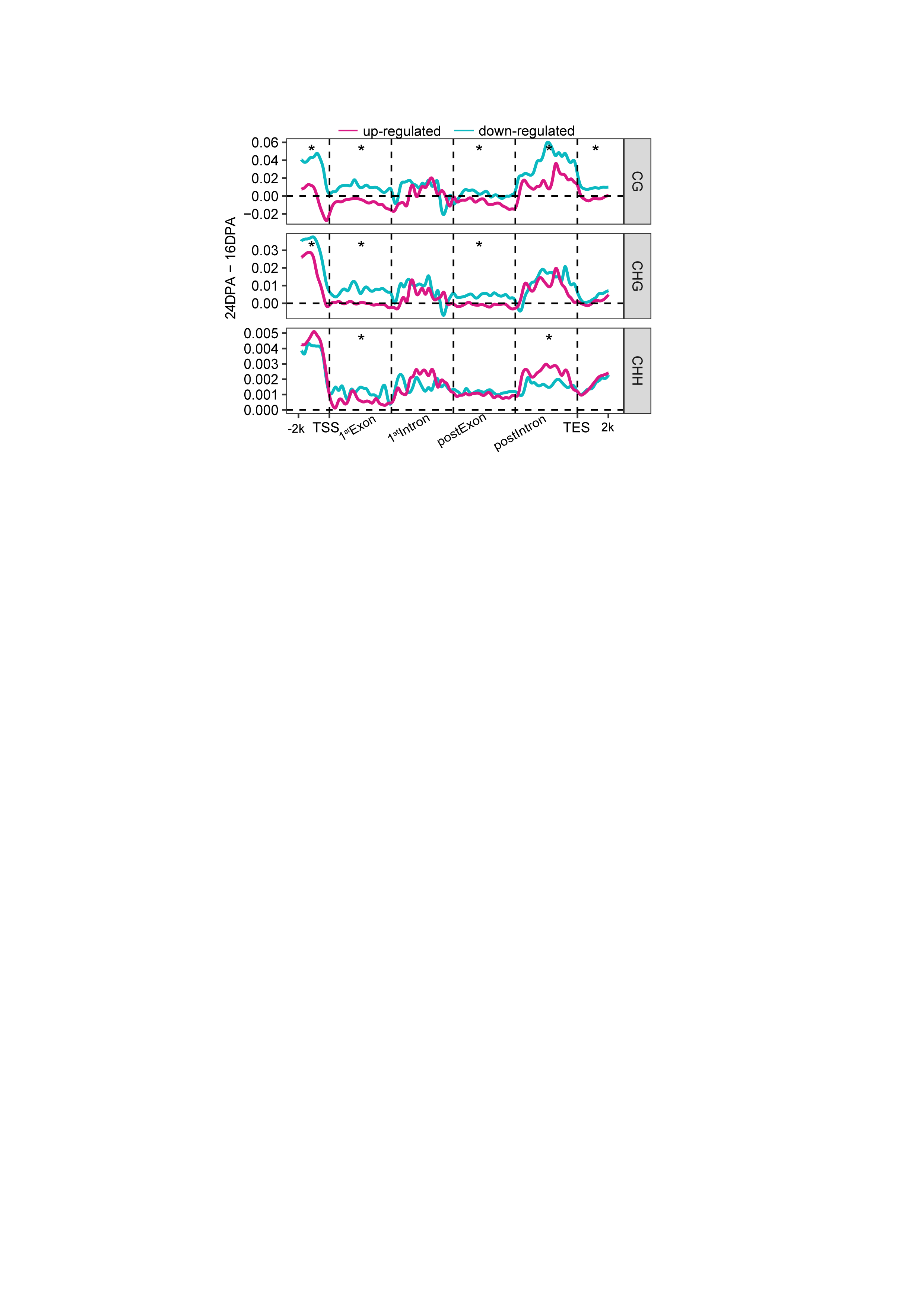
**

**Supplementary Figure 3.** DNA methylation changes of development-induced DEGs in both fields relative to the changes of all genes on gene bodies and flanking regions between 16 and 24 DPA in GY. Red line indicates up-regulated expression genes (FC ≥ 2, FDR < 0.05) and blue line indicates down-regulated expression genes (FC ≤ 0.5, FDR < 0.05). Statistical analysis was done based on paired Student’s *t*-test. *, *P* < 0.01.

**
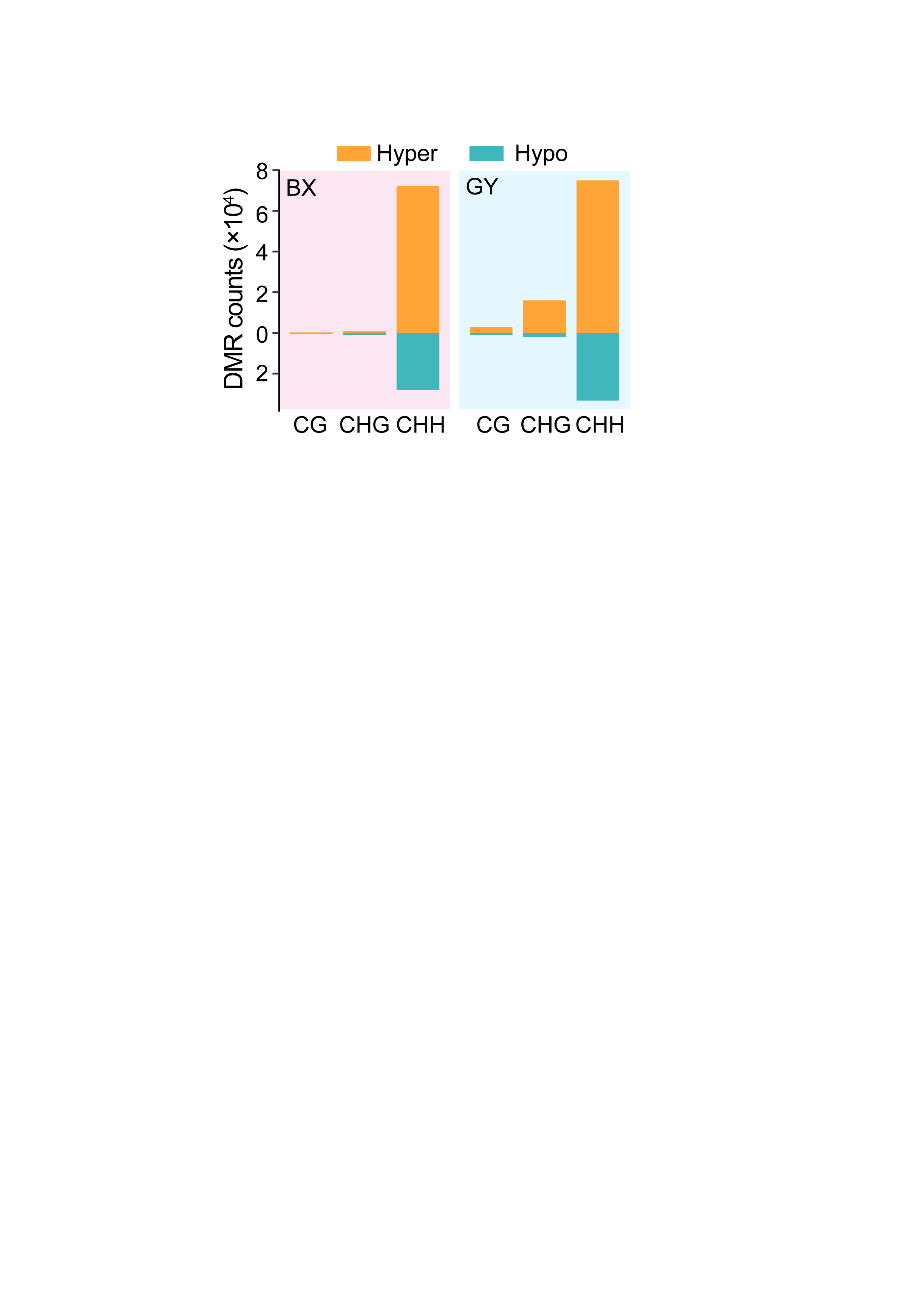
**

**Supplementary Figure 4.** Counts of whole genome DMRs from 16 to 24 DPA in the two fields.

**
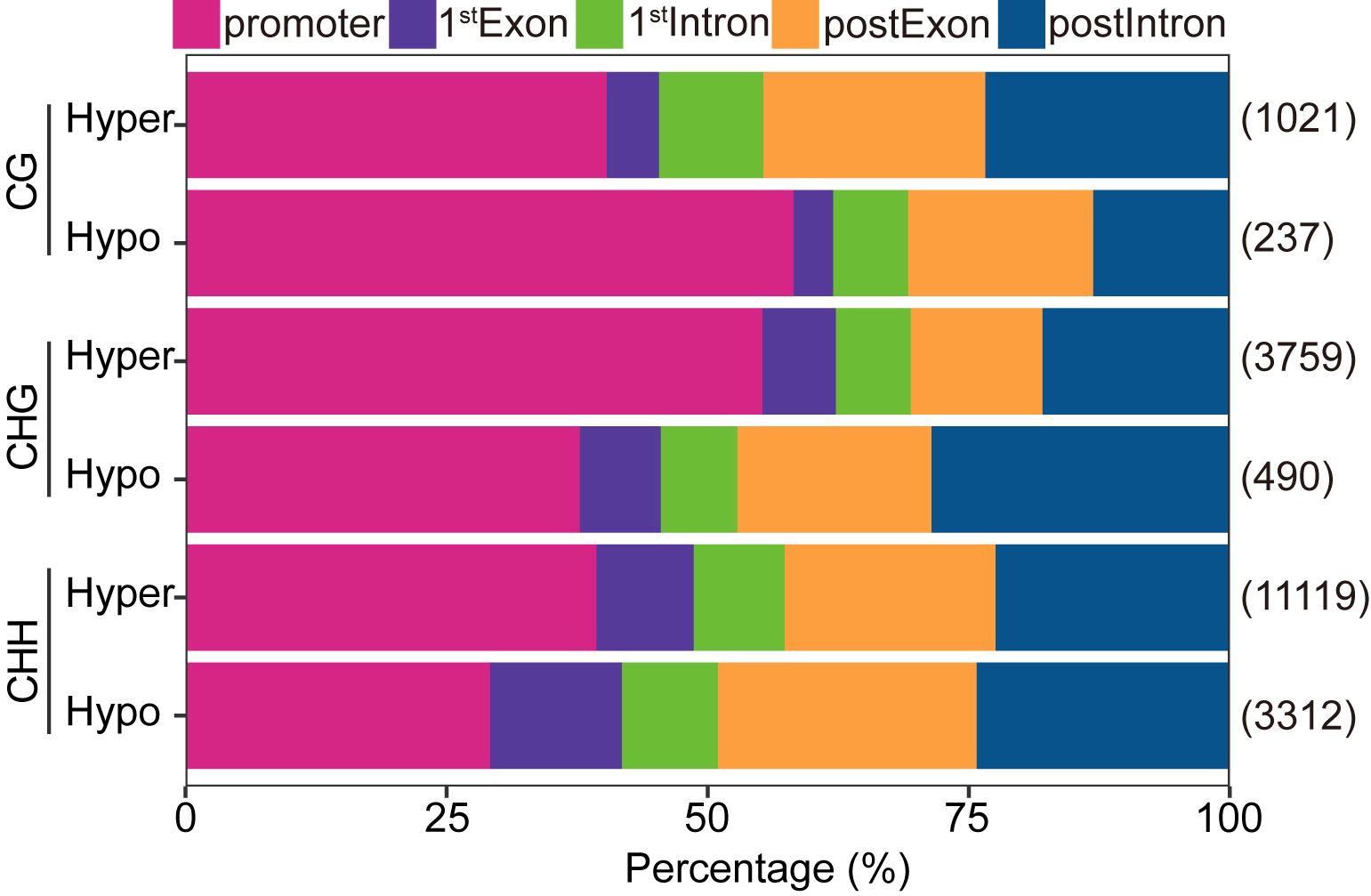
**

**Supplementary Figure 5.** Percentage of DMRs in different functional regions between 16 and 24 DPA in GY. The numbers in parentheses represent the total number of DMRs.

**
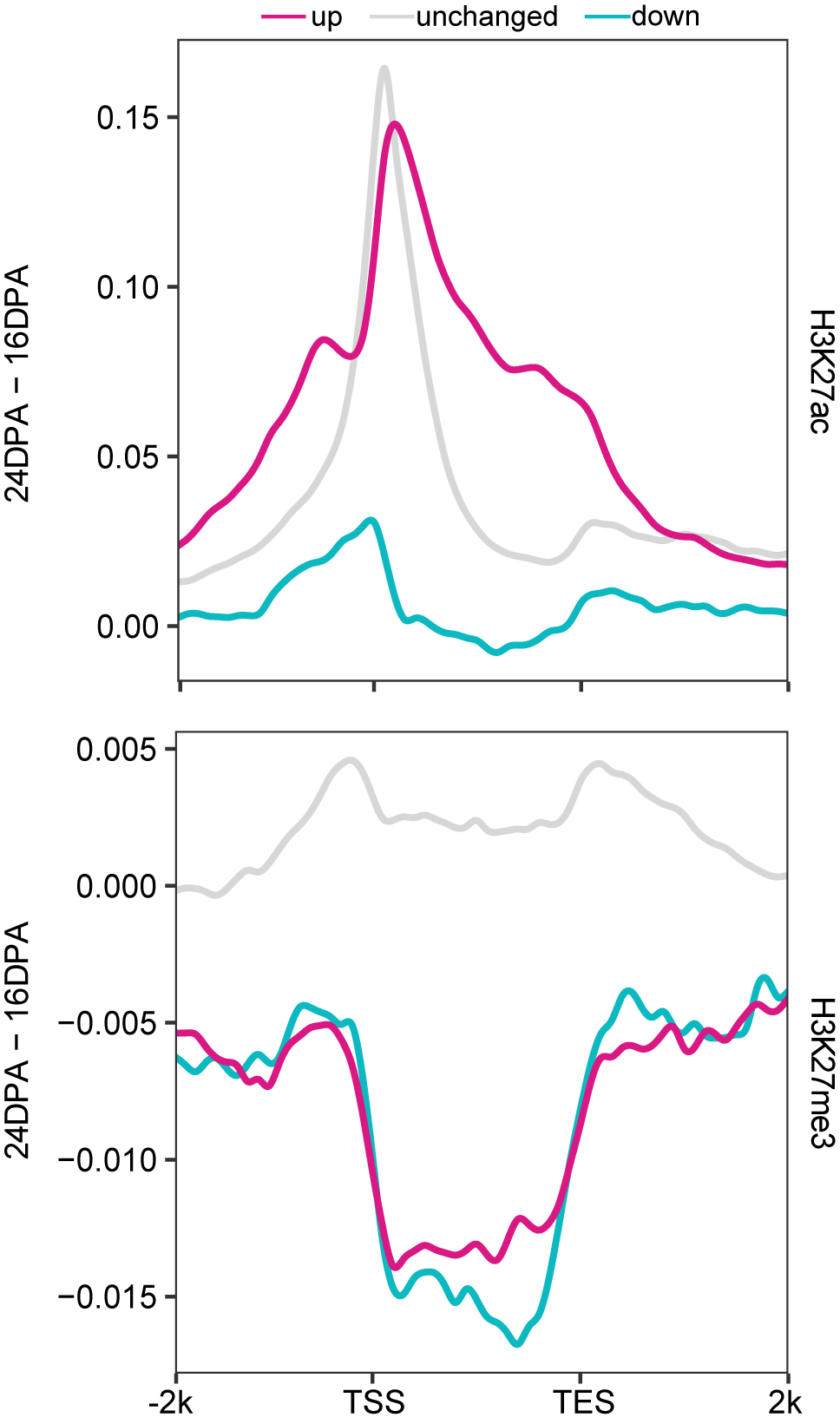
**

**Supplementary Figure 6.**  The intensity variation of H3K27ac and H3K27me3 across gene body and flanking regions of development-induced genes between 16 and 24 DPA in GY. Statistical analysis was done based on paired Student’s *t*-test. *, *P* < 0.01.

**
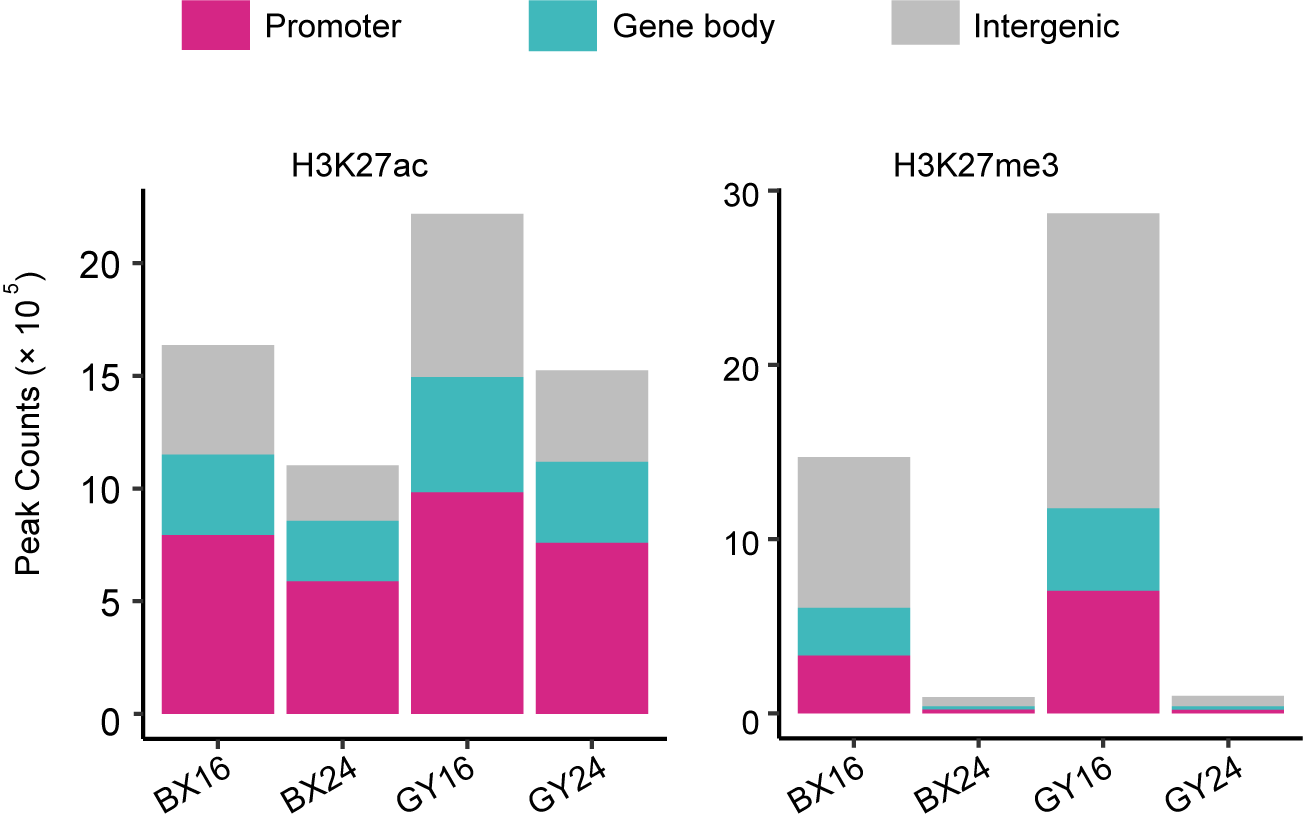
**

**
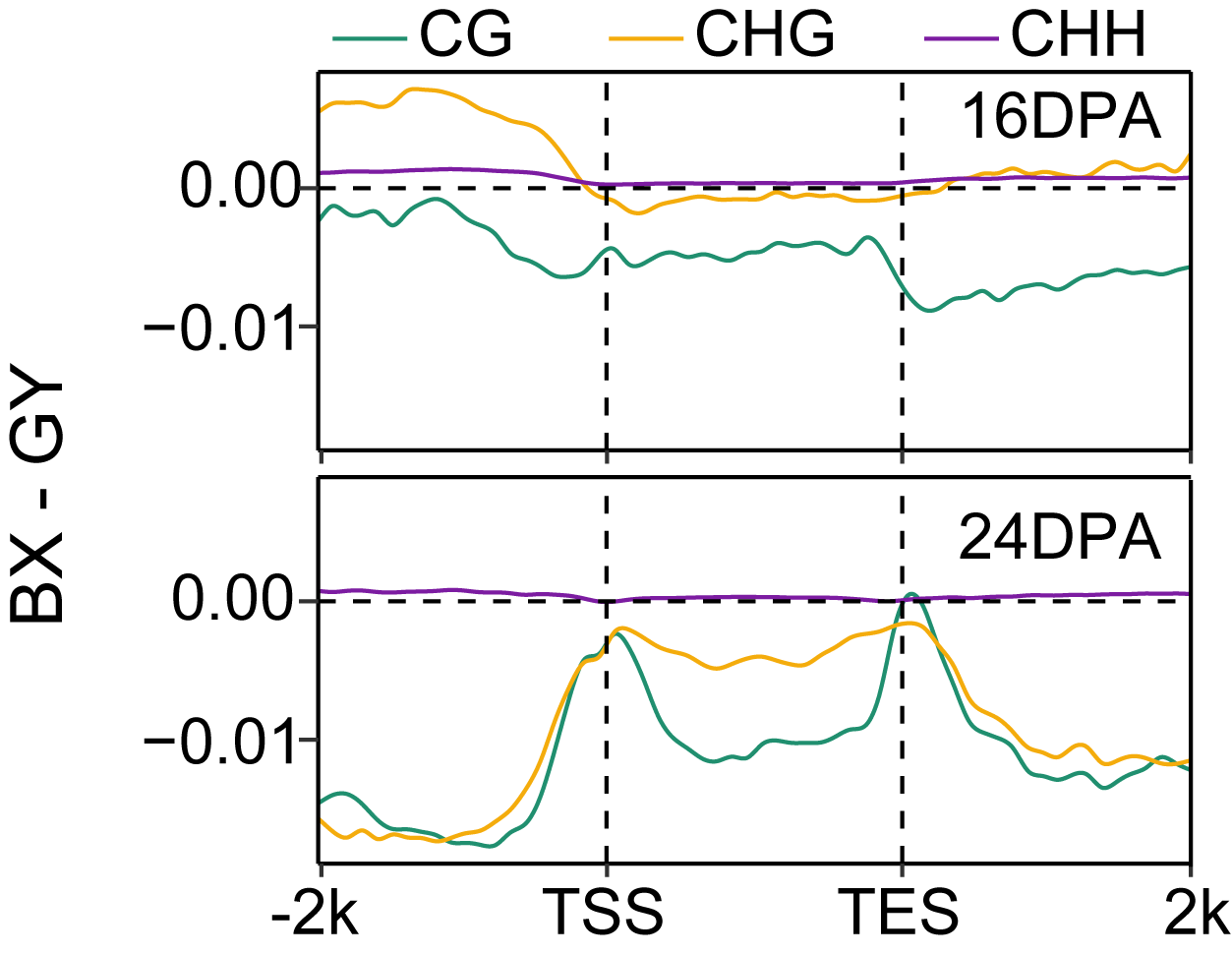
Supplementary Figure 7.** Distribution of differentially modified peaks (DMPs) for H3K27ac and H3K27me3 between 16 and 24 DPA in BX.

**Supplementary Figure 8.** DNA methylation changes in all protein-coding genes between BX and GY at 16 and 24 DPA.

**
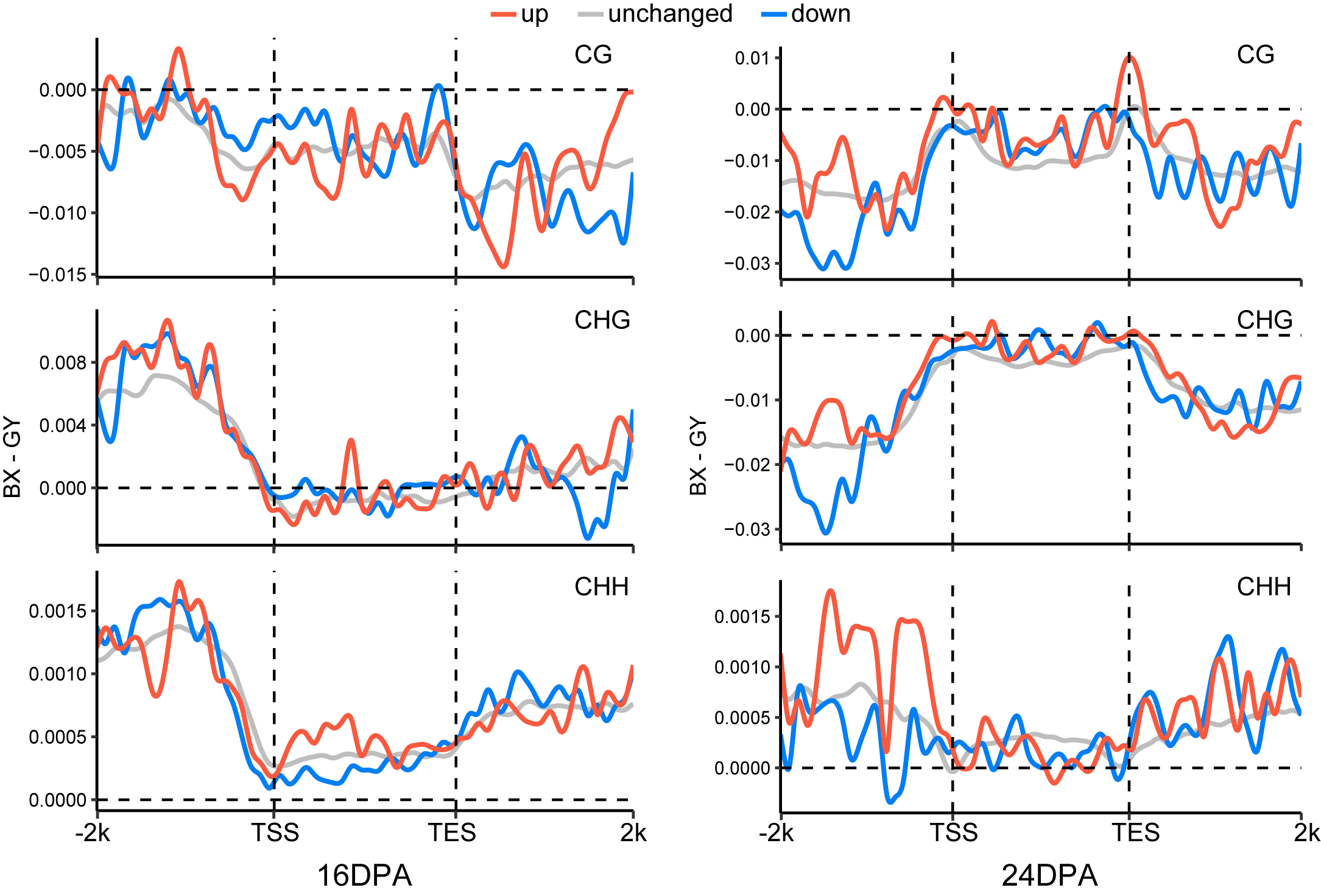
**

**Supplementary Figure 9.** DNA methylation changes of DEGs relative to the changes of all genes on gene bodies and flanking regions between BX and GY at 16 and 24 DPA.

**
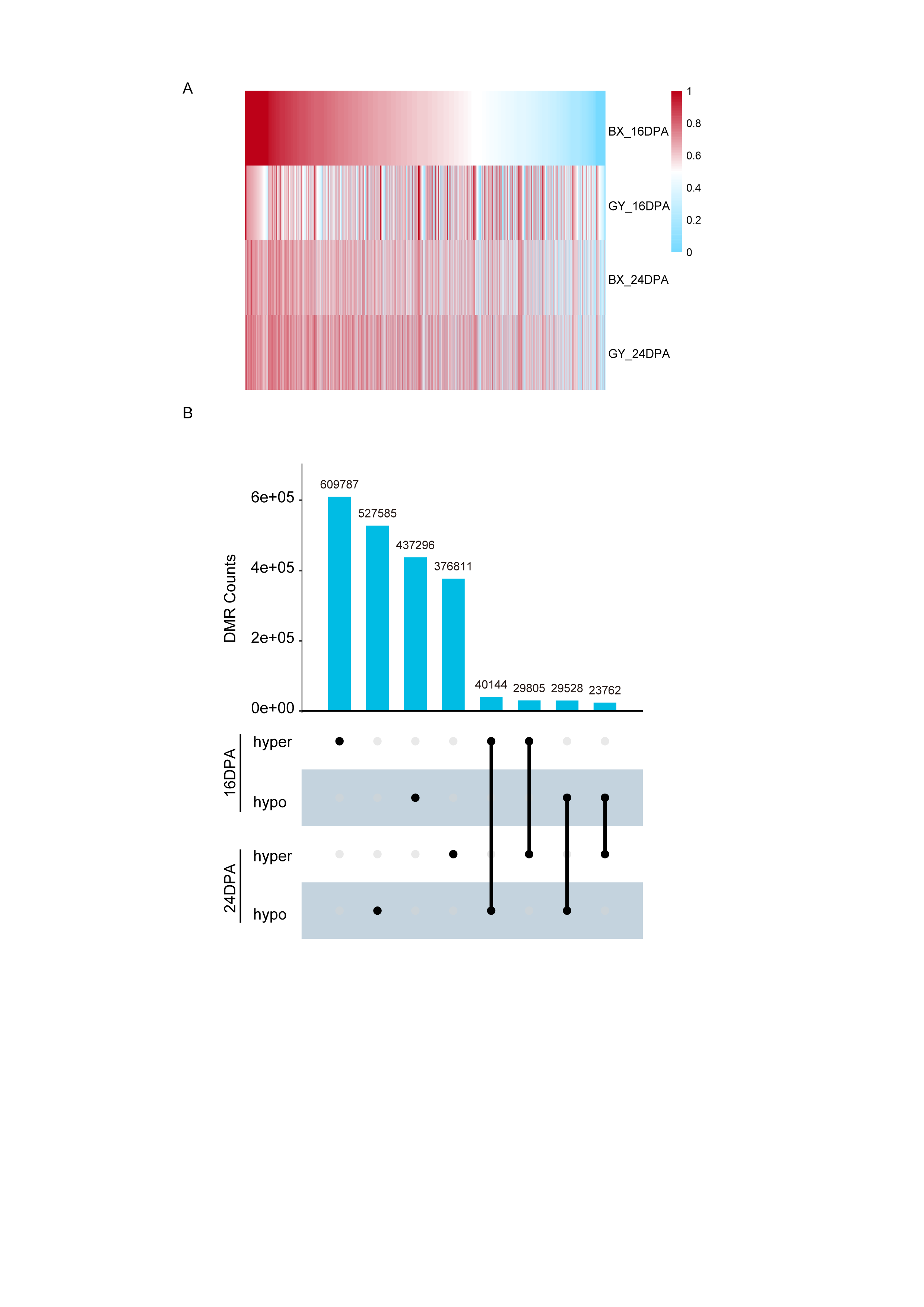
**

**Supplementary Figure 10.** (A) Identified CHG-DMRs at 16 and 24 DPA between BX and GY. (B) Overlap of hypomethylated and hypermethylated CHG-DMRs between BX and GY at 16 and 24 DPA.

**
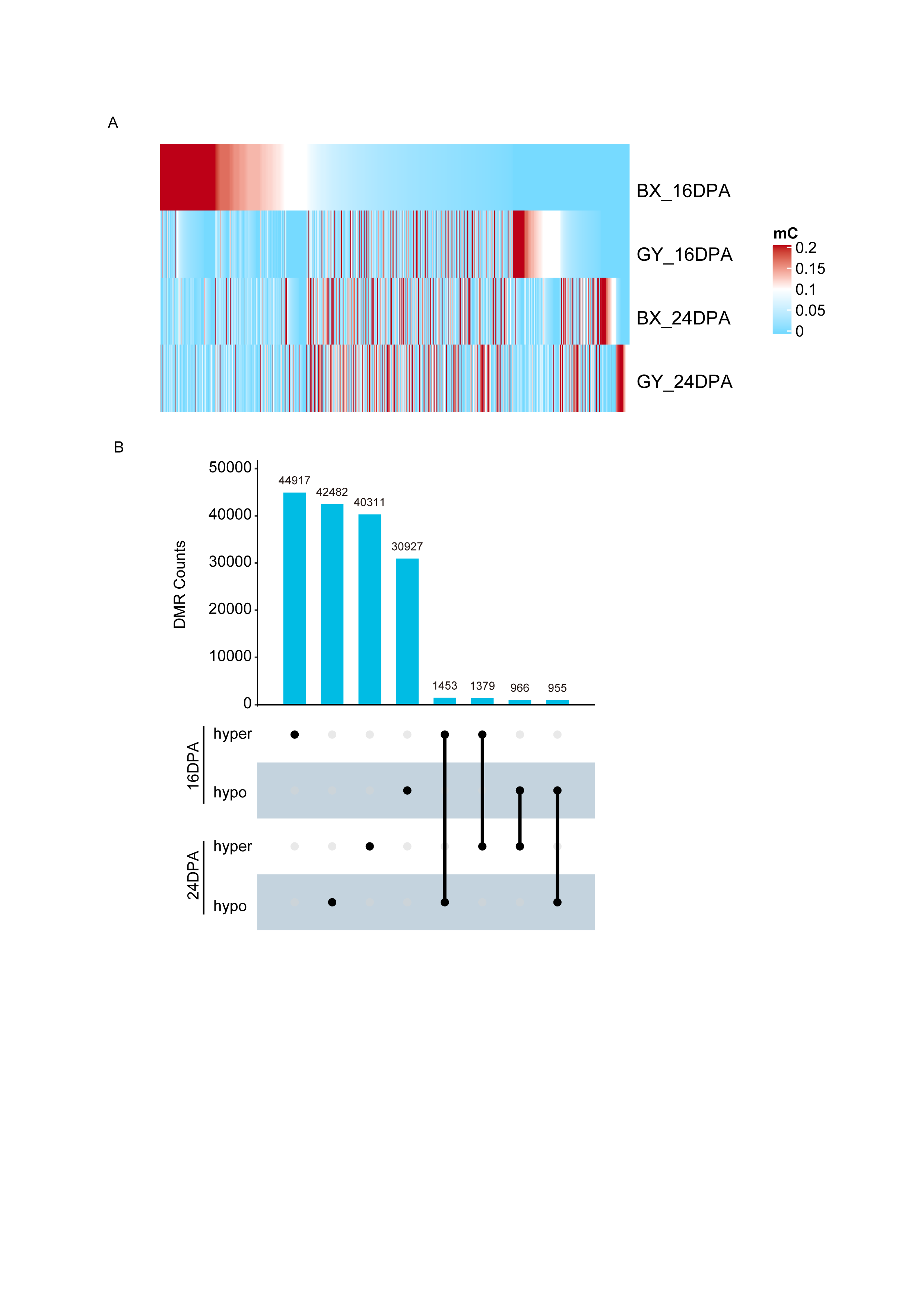
**

**Supplementary Figure 11.** (A) Identified CHH-DMRs at 16 and 24 DPA between BX and GY. (B) Overlap of hypomethylated and hypermethylated CHH-DMRs between BX and GY at 16 and 24 DPA.

**
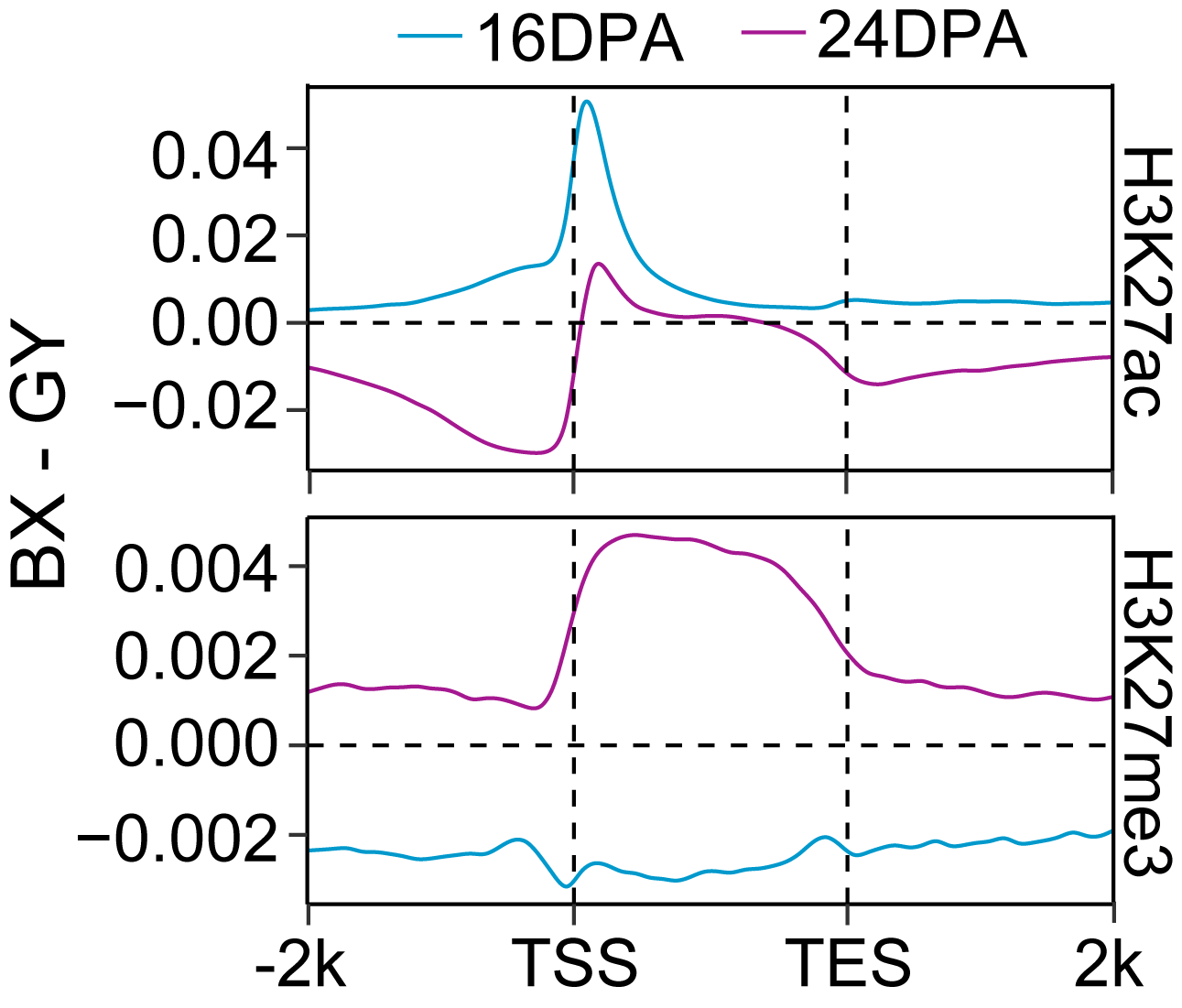
**

**Supplementary Figure 12.** Comparison of the intensity variation of H3K27ac and H3K27me3 on gene bodies and flanking regions between BX and GY at 16 and 24 DPA.

**
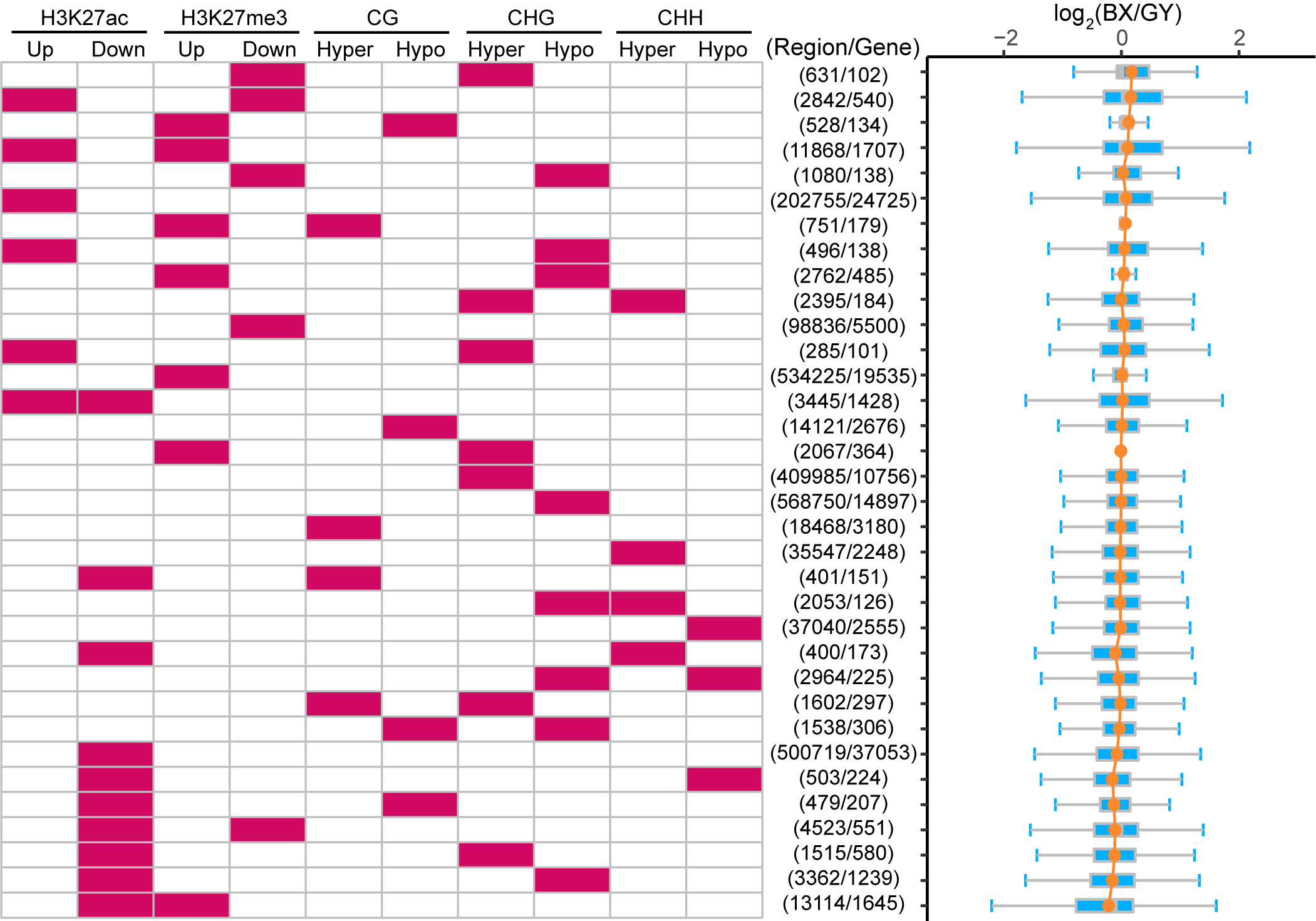
**

**Supplementary Figure 13.** Statistics of epigenetic modification differences between BX and GY across genomic regions at 24 DPA, along with expression changes of overlapping genes, sorted by fold change (log_2_(BX/GY)) from top to bottom.

**
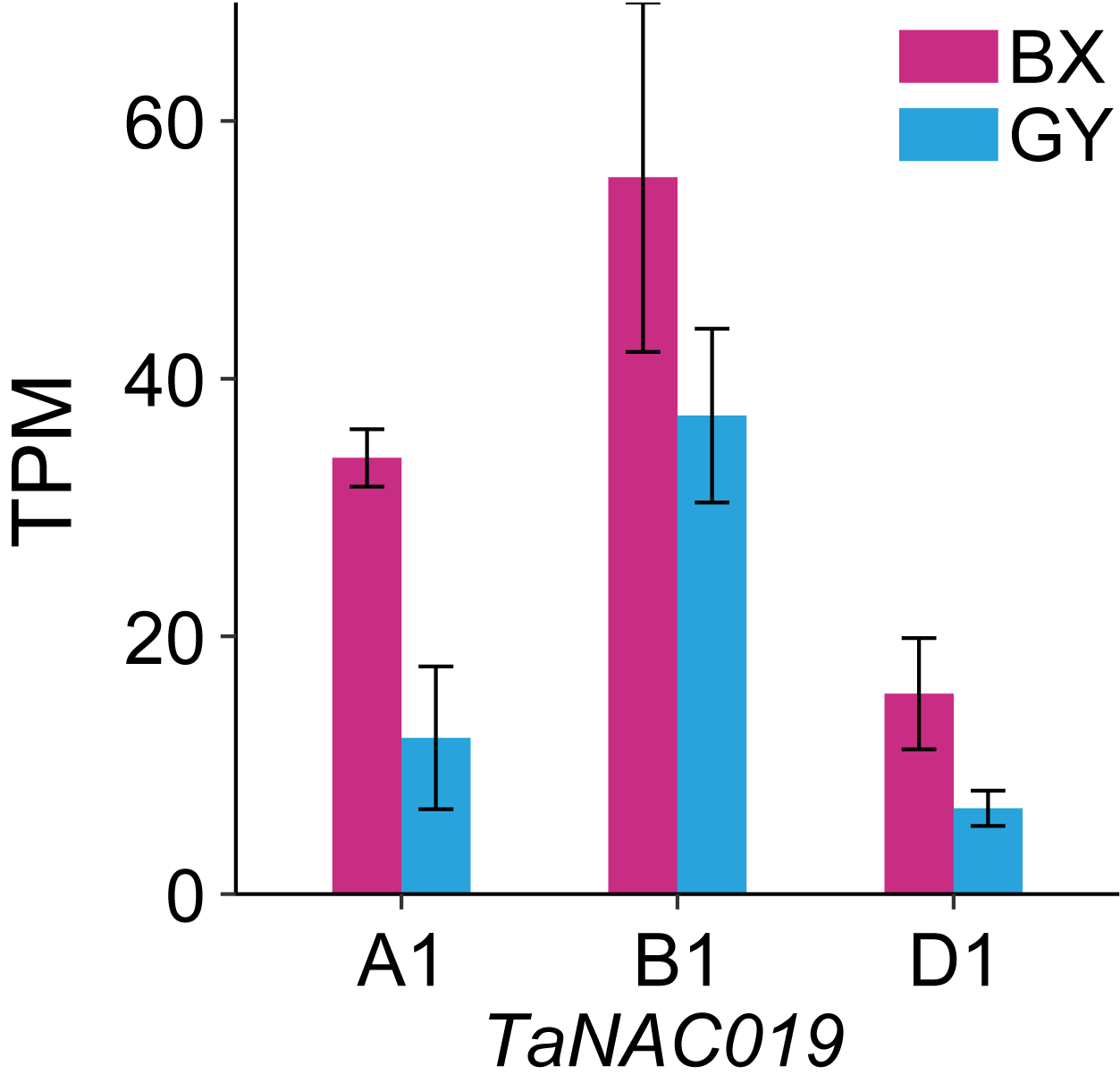
**

**
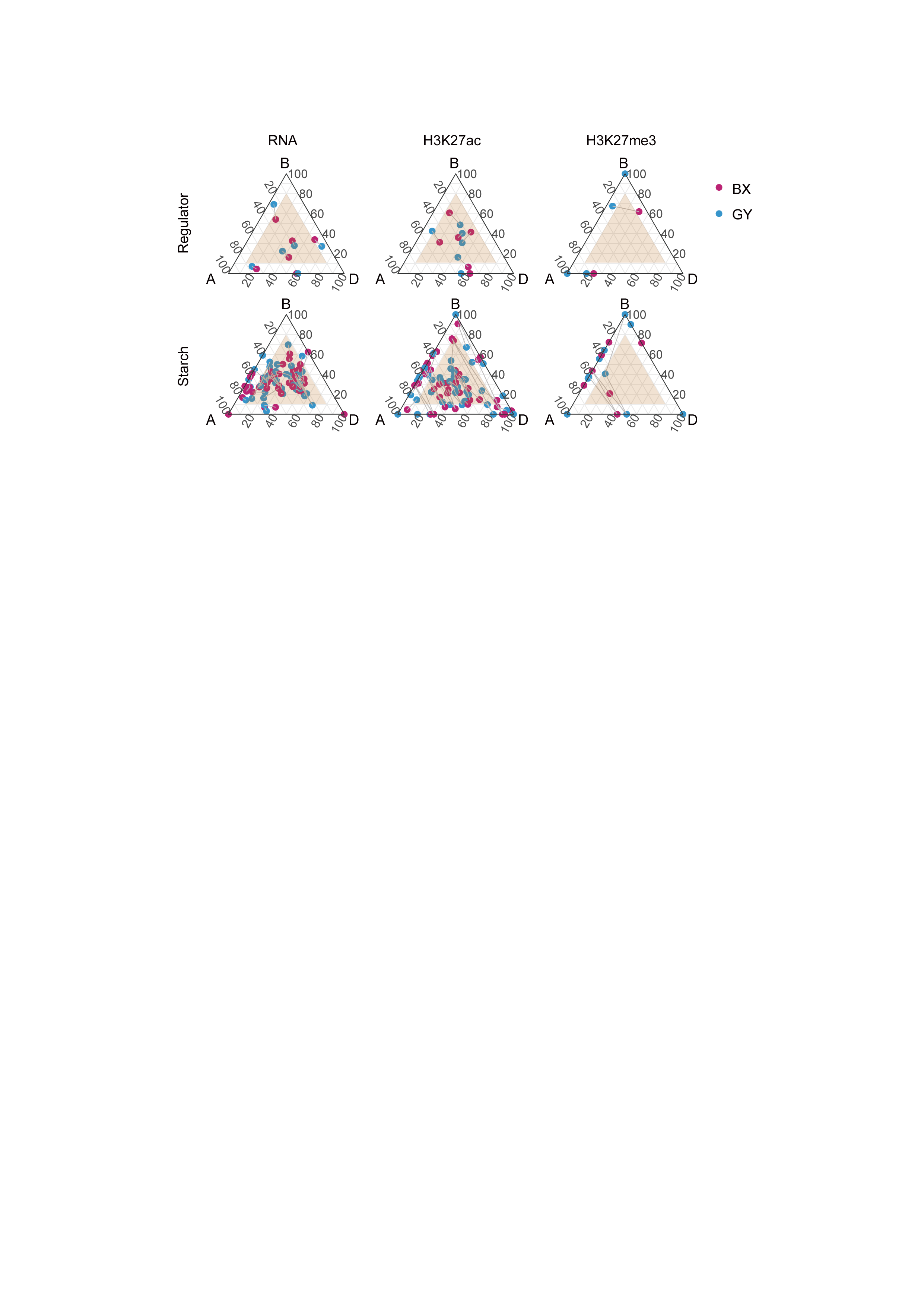
Supplementary Figure 14.** Comparison of the expression of *TaNAC019* homoeologs between BX and GY at 16 DPA.

**Supplementary Figure 15.** Ternary plot showing relative expression and epigenetics modification (H3K27ac and H3K27me3) abundance of regulators and starch biosynthesis genes at 24 DPA. Each circle represents a gene triad with A, B, and D coordinates consisting of the relative contribution of each homoeolog to the overall triad. The dash between each circle represents the same gene triad in BX and GY. Balanced triads are shown within a brown shade.


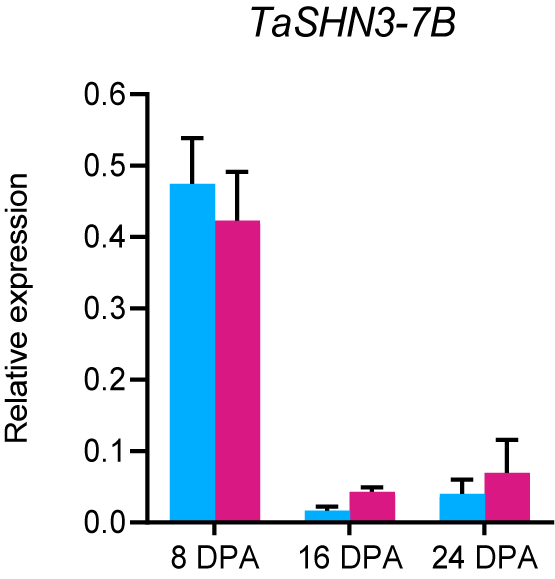


**Supplementary Figure 16.** The relative expression level of *TaSHN3-7B* in 'Fengdecunmai 5' grains at 8, 16, and 24 DPA between BX and GY. qRT-PCR data were normalized to *TaActin*. Statistical significance was determined by two-way ANOVA.
